# SHLD1 is dispensable for 53BP1-dependent V(D)J recombination but critical for productive class switch recombination

**DOI:** 10.1038/s41467-022-31287-3

**Published:** 2022-06-28

**Authors:** Estelle Vincendeau, Wenming Wei, Xuefei Zhang, Cyril Planchais, Wei Yu, Hélène Lenden-Hasse, Thomas Cokelaer, Juliana Pipoli da Fonseca, Hugo Mouquet, David J. Adams, Frederick W. Alt, Stephen P. Jackson, Gabriel Balmus, Chloé Lescale, Ludovic Deriano

**Affiliations:** 1 Institut Pasteur, Université Paris Cité, INSERM U1223, Équipe Labellisée Ligue Contre Le Cancer, Genome Integrity, Immunity and Cancer Unit, 75015 Paris, France; 2 Howard Hughes Medical Institute, Program in Cellular and Molecular Medicine at Boston Children’s Hospital, Department of Genetics, Harvard Medical School, Boston, MA 02115 USA; 3 Institut Pasteur, Université de Paris, INSERM U1222, Laboratory of Humoral Immunology, 75015 Paris, France; 4 Institut Pasteur, Plate-forme Technologique Biomics, Centre de Ressources et Recherches Technologiques, 75015 Paris, France; 5 Institut Pasteur, Hub de Bioinformatique et Biostatistique, Département de Biologie Computationnelle, 75015 Paris, France; 6 Wellcome Trust Sanger Institute, Cambridge, CB10 1SA UK; 7 Wellcome Trust/Cancer Research UK Gurdon Institute, Department of Biochemistry, University of Cambridge, Cambridge, CB2 1QN UK; 8 UK Dementia Research Institute at University of Cambridge, Department of Clinical Neurosciences, University of Cambridge, Cambridge, CB2 0AH UK; 9 Present Address: Biomedical Pioneering Innovation Center (BIOPIC) and Beijing Advanced Innovation Center for Genomics (ICG), Peking University, Beijing, 100871 China

**Keywords:** Class switch recombination, Double-strand DNA breaks, DNA recombination, VDJ recombination

## Abstract

SHLD1 is part of the Shieldin (SHLD) complex, which acts downstream of 53BP1 to counteract DNA double-strand break (DSB) end resection and promote DNA repair via non-homologous end-joining (NHEJ). While 53BP1 is essential for immunoglobulin heavy chain class switch recombination (CSR), long-range V(D)J recombination and repair of RAG-induced DSBs in XLF-deficient cells, the function of SHLD during these processes remains elusive. Here we report that SHLD1 is dispensable for lymphocyte development and RAG-mediated V(D)J recombination, even in the absence of XLF. By contrast, SHLD1 is essential for restricting resection at AID-induced DSB ends in both NHEJ-proficient and NHEJ-deficient B cells, providing an end-protection mechanism that permits productive CSR by NHEJ and alternative end-joining. Finally, we show that this SHLD1 function is required for orientation-specific joining of AID-initiated DSBs. Our data thus suggest that 53BP1 promotes V(D)J recombination and CSR through two distinct mechanisms: SHLD-independent synapsis of V(D)J segments and switch regions within chromatin, and SHLD-dependent protection of AID-DSB ends against resection.

## Abstract (web summary)

SHLD1, as a component of the Shieldin (SHLD) complex, mediates DNA repair via non-homologous end-joining (NHEJ), an essential process during lymphocyte development. Here the authors show that SHLD1 is actually dispensable for lymphocyte development and V(D)J recombination, but is essential for class-switching recombination in activated B cells.

## Introduction

Two programmed DNA double-strand break (DSB) repair processes participate in the generation of antigen receptor diversity in adaptive immune cells—V(D)J recombination that occurs in developing B and T lymphocytes and class switch recombination (CSR) that takes place in antigen-activated mature B cells^1^. V(D)J recombination is a cut and paste mechanism that assembles variable region exons encoding the antigen-binding domains of the B and T cell receptors^2,3^. It is initiated by recombination activating gene proteins RAG1 and RAG2 (forming the RAG endonuclease) which introduce DSBs between variable (V), diversity (D), and joining (J) segments and their flanking recombination signal sequences (RSSs)^4^. RAG-generated DSBs are subsequently repaired by proteins of the non-homologous end-joining (NHEJ) pathway within a so-called post-cleavage complex that is thought to participate in the stabilization and processing of DNA ends^5,6^. CSR modifies antibody effector functions by replacing the isotype expressed from IgM/IgD to IgG, IgA, or IgE^1,7,8^. At the DNA level, this is achieved by a deletional recombination event at the *Igh* locus between a donor (Sμ) and an acceptor repetitive switch region (Sx) that brings into proximity the V region and the exons encoding a new constant region thus allowing the expression of an antibody with a different isotype but with the same specificity. During CSR, activation-induced cytidine deaminase (AID) deaminates cytosines into uracils in transcribed S-regions, which are converted to DSBs with the help of base excision and mismatch repair proteins. CSR is completed by fusing donor and acceptor S-region DSB ends by NHEJ and, in its absence, by alternative end-joining (alt-NHEJ) that is more biased to use longer junctional microhomologies (MHs)^9,10^. In contrast to CSR, V(D)J recombination is much more reliant on NHEJ for the repair of RAG-DSBs and NHEJ-deficient mice—with the exception of XLF-deficient mice—suffer severe block in early B and T cell differentiation due to an inability to assemble V(D)J segments and thus to produce a functional pre-T or pre-B cell receptor^9,10^.

Both RAG- and AID-induced DSBs trigger a DNA damage response during which DSB ends are detected by the MRE11-RAD50-NBS1 complex that activates ataxia telangiectasia-mutated kinase (ATM)^1,11,12^. Once activated, ATM phosphorylates multiple substrates including the histone variant H2AX that serves as an intermediate step to promote the assembly of DNA repair proteins over large chromatin regions surrounding the break site, most notably 53BP1 that can be visualized as chromatin forming foci in recombining lymphocytes^1,11,12^. Unlike NHEJ factors, these proteins are essential for CSR and have a more modest role in V(D)J recombination^1,11,12^. For instance, ATM-deficient cells are still capable of V(D)J recombination and ATM-deficient mice are only moderately immune-deficient but activated ATM-deficient splenic B cells have impaired CSR (i.e., 30 to 50% of wild-type (WT) cells)^1,11,12^. More illustrative is the case of 53BP1. In mice, 53BP1 deficiency has a modest impact on lymphocyte development and V(D)J recombination but causes a drastic reduction in CSR (i.e., 5 to 10% of WT cells) that is associated with enhanced intra S-region recombination, long DSB end resection that spreads outside S-regions and loss of orientation-specific recombination^11,13–17^. This suggests specialized roles for 53BP1 in CSR such as promoting S-region synapsis and protecting S-region DSBs from resection.

The absence of a striking phenotype during lymphocyte development and V(D)J recombination in animals deficient for these DSB response factors does not mean that they have no role at all in promoting RAG-DSB repair. 53BP1 facilitates the joining of RAG-induced DSBs when DSBs are at a long distance, possibly by favoring chromosome tethering and/or mobility^18^. In the absence of XLF, a nonessential NHEJ factor, ATM and 53BP1 are required for repair of RAG-generated coding ends (CEs) and signal ends (SEs) and double-deficient mice have a severe combined immunodeficiency phenotype with lymphocyte development essentially blocked at the progenitor B and T cell stages, consistent with impaired V(D)J recombination^19–22^. Analyses of XLF/53BP1 double-deficient Abelson (Abl)-immortalized pro-B cell lines revealed a combined impact on the ability to join RAG-DSBs during V(D)J recombination, including the impaired joining of both CEs and SEs and increased resection of the un-joined CEs and SEs^19–22^. Thus, in the context of the ATM-dependent DSB response, 53BP1 seems to harbor similar DSB end-joining and DSB end-protection functions during CSR as well as during V(D)J recombination.

53BP1 recruits RIF1 to shield DSB ends against resection, thereby directing the repair of DSBs toward NHEJ instead of homologous recombination^23^. The downstream effectors in the pathway have recently been identified and form the Shieldin (SHLD) complex that lies immediately downstream of 53BP1-RIF1 and is comprised of REV7/MAD2L2, SHLD1, SHLD2, and SHLD3^23–25^. Epistasis between 53BP1-RIF1 and the SHLD complex is remarkable in multiple NHEJ reactions, including CSR, the fusion of telomeres lacking TRF2, and the repair of DSBs in BRCA1-deficient cells treated with PARP inhibitors^23–25^. In AID-inducible B cell lines, loss of any of the SHLD complex components similarly impairs CSR that is associated with enhanced DSB end resection and the accumulation of chromosomal breaks at immunoglobulin switching sites^26–34^. In mice, loss of REV7 or SHLD2 does not impact lymphocyte development while severely compromising CSR^27,30^. The recruitment of SHLD to DSBs is mediated by the association of SHLD3 to chromatin-bound RIF1^25^. SHLD3 also interacts directly with REV7 that bridges SHLD2 and SHLD1, with SHLD2 possessing a ssDNA-binding activity that is essential to inhibit resection^25^. SHLD1 is a small (205 amino acid residues) low-abundance protein that lies at the very tip of the complex and for which clear functions have not yet been attributed^25^.

Here, we generate mice and cells deficient for SHLD1, XLF, and XRCC4 to test the role of SHLD1 in antigen receptor diversification. We show that SHLD1- and SHLD1/XLF-deficient mice have normal lymphocyte development and perform robust RAG-DSB repair, demonstrating that SHLD1 is dispensable for V(D)J recombination. By contrast, we show that SHLD1 is essential to limit the extent of DNA end resection during CSR, providing an end-protection mechanism that enables both NHEJ and alternative end-joining pathways to functionally operate at AID-induced DSBs. Finally, we show that this end-protection function is required for the orientation-specific joining of AID-initiated DSBs. Thus, while SHLD1 is dispensable for V(D)J recombination, it is essential for productive CSR by controlling the processing of AID-DNA ends during NHEJ and alternative end-joining repair.

## Results

### *Shld1*^*−/−*^ and *Shld1*^*−/−*^*Xlf*^*−/−*^ mice are viable and show no overt developmental phenotype

**Fig. 1:**
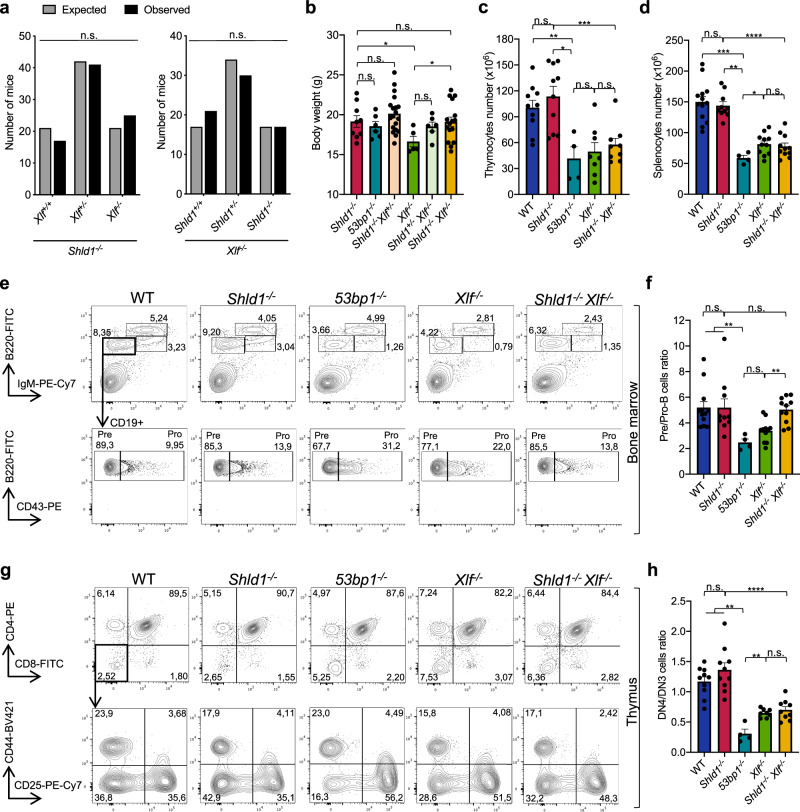
*Shld1*^*−/−*^ and *Shld1*^*−/−*^*Xlf*^*−/−*^ mice are viable and show no overt developmental phenotype. **a** Number of live-born mice obtained from crosses between *Shld1*^*−/−*^ *Xlf*^*+/−*^ mice (left) or *Shld1*^*+/−*^ *Xlf*^*−/−*^ mice (right). Expected versus observed numbers were used to calculate one-sided Chi-square. *Shld1*^*−/−*^ *Xlf*^*+/−*^ cross: *n* = 83 observed live-born mice; *Shld1*^*+/−*^ *Xlf*^*−/−*^ cross: *n* = 68 observed live-born mice. **b** Total body weight (in grams) of adult mice (males and females, 6–8-week-old) from the different genotypes as indicated. Bars represent mean ± s.e.m., *n* = 9 independent mice (*Shld1*^*−/−*^), *n* = 6 (*53bp1*^*−/−*^), *n* = 19 (*Shld1*^*−/−*^ *Xlf*^*+/−*^), *n* = 5 (*Xlf*^*−/−*^), *n* = 6 (*Shld1*^*+/−*^ *Xlf*^*−/−*^), *n* = 16 (*Shld1*^*−/−*^ *Xlf*^*−/−*^), two-sided Wilcoxon–Mann–Whitney test (*p = 0.047 (*Xlf*^*−/−*^ vs *Shld1*^*−/−*^); *p = 0.0268 (*Xlf*^*−/−*^ vs *Shld1*^*−/−*^ *Xlf*^*−/−*^)). **c** Total thymocytes numbers. Bars represent mean ± s.e.m., *n* = 10 (WT; *Shld1*^*−/−*^), *n* = 4 (*53bp1*^*−/−*^), *n* = 8 (*Xlf*^*−/−*^), *n* = 9 (*Shld1*^*−/−*^ *Xlf*^*−/−*^), two-sided Wilcoxon–Mann–Whitney test (*p = 0.014; **p = 0.008; ***p = 0.0005). WT: dark blue, *Shld1*^*−/−*^: red, *53bp1*^*−/−*^: light blue, *Xlf*^*−/−*^: green, *Shld1*^*−/−*^*Xlf*^*−/−*^: yellow. **d** Total splenocytes numbers. Bars represent mean ± s.e.m., *n* = 13 (WT), *n* = 10 (*Shld1*^*−/−*^), *n* = 4 (*53bp1*^*−/−*^), *n* = 11 (*Xlf*^*−/−*^; *Shld1*^*−/−*^ *Xlf*^*−/−*^), two-sided Wilcoxon–Mann–Whitney test (*p = 0.0396; **p = 0.002; ***p = 0.0008; ****p < 0.0001). **e**, **f** Analysis of B cell development. **e** Representative FACS analysis of bone marrow using B cell markers. **f** Ratio of CD43^−^B220^lo^CD19^+^IgM^−^ pre-B cells versus CD43^+^B220^lo^CD19^+^IgM^−^ pro-B cells. Bars represent mean ± s.e.m., *n* = 12 (WT), *n* = 10 (*Shld1*^*−/−*^), *n* = 4 (*53bp1*^*−/−*^), *n* = 11 (*Xlf*^*−/−*^; *Shld1*^*−/−*^ *Xlf*^*−/−*^), two-sided Wilcoxon–Mann–Whitney test (**p = 0.0014 (*Xlf*^*−/−*^ vs *Shld1*^*−/−*^ *Xlf*^*−/−*^); **p = 0.0011 (WT vs *53bp1*^*−/−*^); **p = 0.004 (*Shld1*^*−/−*^ vs *53bp1*^*−/−*^)). **g**, **h** Analysis of T cell development. **g** Representative FACS analysis of thymus using T cell markers. **h** Ratio of CD4^−^CD8^−^CD44^−^CD25^−^ (DN4) cells versus CD4^−^CD8^−^CD44^−^CD25^+^ (DN3) cells. Bars represent mean ± s.e.m., *n* = 10 (WT; *Shld1*^*−/−*^), *n* = 4 (*53bp1*^*−/−*^), *n* = 8 (*Xlf*^*−/−*^), *n* = 9 (*Shld1*^*−/−*^ *Xlf*^*−/−*^), two-sided Wilcoxon–Mann–Whitney test (**p = 0.004 (*Xlf*^*−/−*^ vs *53bp1*^*−/−*^); **p = 0.002 (WT vs *53bp1*^*−/−*^); **p = 0.002 (*Shld1*^*−/−*^ vs *53bp1*^*−/−*^); ****p < 0.0001 (*Shld1*^*−/−*^ vs *Shld1*^*−/−*^ *Xlf*^*−/−*^)). n.s. non-significant (*p* ≥ 0.05), *p < 0.05, **p < 0.01, ***p < 0.001, ****p < 0.0001. Source data are provided as a Source Data file.

To elucidate SHLD1 function in vivo, we analyzed *Shld1* knockout mice (*Shld1*^*em1(IMPC)Wtsi*^, referred to hereafter as *Shld1*^*−/−*^) that we generated by CRISPR/Cas9-mediated deletion of the *Shld1* coding sequence^35–37^ (Supplementary Fig. 1a–d). *Shld1*^*−/−*^ mice were born at the expected Mendelian frequencies, were fertile and displayed no developmental abnormalities (Fig. 1a, b and Supplementary Fig. 1e, f).

Total cell numbers in the thymus and spleen of 6- to 8-week-old *Shld1*^*−/−*^ mice were, on average, similar to those of wild-type (WT) (Fig. 1c, d). Flow cytometry revealed that the distribution of progenitor (pro-) B (CD19^+^CD43^+^B220^+^IgM^−^), precursor (pre-) B (CD19^+^CD43^−^B220^+^IgM^−^), newly-generated immature B (IgM^+^B220^low^) and recirculating B (IgM^+^B220^high^) cells in *Shld1*^*−/−*^ bone marrow were similar to those of WT littermates (Fig. 1e and Supplementary Table 1). In addition, *Shld1*^*−/−*^ mice contained splenic CD19^+^IgM^+^ total B cells, Follicular (B220^+^CD93^−^CD23^+^CD21^+^) and Marginal Zone (B220^+^CD93^−^CD23^−^CD21^high^) B cells at distributions and numbers equivalent to WT (Supplementary Fig. 1g–i and Supplementary Table 2). Similarly, the distribution of CD4^−^CD8^−^ (double-negative, DN), CD4^+^CD8^+^ (double-positive, DP), and CD4^+^CD8^−^ or CD4^−^ CD8^+^ (single-positive, SP) *Shld1*^*−/−*^ thymocytes was comparable to those of WT littermates (Fig. 1g and Supplementary Table 3) and *Shld1*^*−/−*^ mice contained normal populations of splenic CD3^+^TCRβ^+^ T cells (Supplementary Fig. 1j and Supplementary Table 2). The absence of B- and T cell developmental block at the CD43^+^B220^+^IgM^−^ pro-B cell (Fig. 1f) and CD4^−^CD8^−^CD44^−^CD25^+^ DN3 thymocyte (Fig. 1h) stages during which antigen receptor gene assembly is initiated suggests that V(D)J recombination is normal in *Shld1*^*−/−*^ developing lymphocytes. Contrary to SHLD1-deficient mice and consistent with previous work^16,18,30^, *53bp1*^*−/−*^ mice harbored impaired B and T cell development, with a moderate block at the CD43^+^B220^+^IgM^−^ pro-B (Fig. 1e, f) and CD4^−^CD8^−^CD44^−^CD25^+^ DN3 pro-T stages (Fig. 1g, h), accompanied by a mild decrease in the number of splenic B and T cell numbers (Supplementary Fig. 1g, j).

XLF is functionally redundant with multiple members of the DNA damage response that include ATM, H2AX and 53BP1^19^. As such, combined deficiency for H2AX and XLF leads to early embryonic lethality and *Atm*^*−/−*^ *Xlf*^*−/−*^ and *53bp1*^*−/−*^ *Xlf*^*−/−*^ mice are born significantly smaller than control littermates and display a severe block in B and T cell development due to an inability to perform V(D)J recombination^20–22^. To test whether SHLD1 has overlapping functions with XLF during mouse development, we bred our *Shld1*^*−/−*^ mice with *Xlf*^*−/−*^ mice^38,39^ to generate *Shld1*^*−/−*^ *Xlf*^*−/−*^ animals (Supplementary Fig. 1b, d). *Shld1*^*−/−*^ *Xlf*^*−/−*^ mice were born at the expected Mendelian frequencies (Fig. 1a) and did not display growth defects (Fig. 1b and Supplementary Fig. 1f), indicating that in contrast to ATM, H2AX, or 53BP1, SHLD1 is dispensable for overall mammalian development in the absence of XLF.

To test for a potential functional redundancy between SHLD1 and XLF during lymphocyte development, we analyzed lymphocyte differentiation in *Shld1*^*−/−*^ *Xlf*^*−/−*^ mice. Strikingly, absolute numbers of thymocytes, total splenocytes as well as splenic B cells and T cells in *Shld1*^*−/−*^ *Xlf*^*−/−*^ mice were comparable to those of *Xlf*^*−/−*^ mice (Fig. 1c, d, Supplementary Fig. 1g, j, and Supplementary Tables 2, 3). In addition, in *Shld1*^*−/−*^ *Xlf*^*−/−*^ mice, there was no exacerbated block in B cell development in the bone marrow and T cell development in the thymus, particularly at pro-B or DN3 populations in which V(D)J recombination occurs (Fig. 1e–h and Supplementary Tables 1, 3). Together, these results show that SHLD1 is dispensable for lymphocyte development in the sensitized XLF-deficient background.

### 53BP1 displays SHLD1-independent functions during V(D)J recombination

**Fig. 2:**
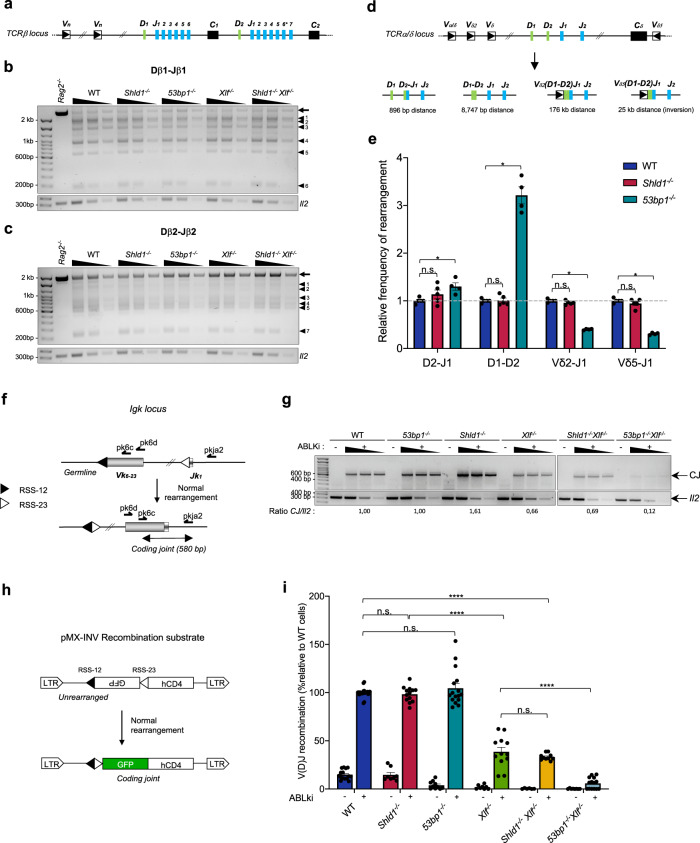
53BP1 displays SHLD1-independent functions during V(D)J recombination. **a** Schematic representation of the murine *Tcrβ* locus. Variable (V), diversity (D), joining (J), and constant (C) segments are shown. The star marks a pseudogene (Jβ2.6). **b** PCR analysis of Dβ1 to Jβ1 rearrangements in *Rag2*^*−/−*^, WT, *Shld1*^*−/−*^, *53bp1*^*−/−*^, *Xlf*^*−/−*^, and *Shld1*^*−/−*^*Xlf*^*−/−*^ thymocytes. The arrows indicate the region in germline configuration. The bands marked by arrowheads represent rearrangements of Dβ to one of the Jβ segments. *Il2* gene PCR was used as a loading control. **c** PCR analysis of Dβ2 to Jβ2 rearrangements. **d** Schematic representation of the mouse *Tcrα/δ* locus with the four different *Tcrδ* rearrangements depicted individually below. **e** Relative frequency of *Tcrδ* locus rearrangements in total thymocytes from WT*, Shld1*^*−/−*^ *and 53bp1*^*−/−*^ mice. Quantitative assessment of genomic DNA rearrangements of Dδ1 to Dδ2, Dδ2 to Jδ1, and Vδ2 and Vδ5 to (D)Jδ1 genes were performed by qPCR and normalized to the signal of the non-rearranging DNA 3′ of Jδ2. Histograms represent the average relative to WT mice for each rearrangement ± s.e.m., *n* = 5 independent samples (except *53bp1*^*−/−*^ for which *n* = 4), two-sided Wilcoxon–Mann–Whitney test (*p = 0.0179). **f** Schematic of inversional *Igk*_*6-23*_ *–J*_*1*_ rearrangement (position of primers (arrows) used to assay coding joint are shown). **g** Semi-quantitative nested PCR analysis of *Igk*_*6-23*_ *–J*_*1*_ coding joint (CJ) in *v-Abl* pro-B cell lines treated for 72 h with ABLki. *Il2* gene PCR was used as a loading control. (WT: clone #12095; *53bp1*^*−/−*^: clone #9999; *Shld1*^*−/−*^: clone #O37; *Xlf*^*−/−*^: clone #16488; *Shld1*^*−/−*^*Xlf*^*−/−*^: clone #XO2-8; *Xlf*^*−/−*^*53bp1*^*−/−*^: clone #9X2). CJ/Il2 ratio of band intensity is indicated below the gel. **h** Schematic of pMX-INV recombination substrate. **i** Bar plot showing V(D)J recombination efficiency of pMX-INV recombination substrate in *v-Abl* pro-B cell lines. *v-Abl* pro-B cell lines of each genotype were treated with ABLki for 72 h and GFP^+^ cells were analyzed by flow cytometry. Bars represent mean ± s.e.m., *n* = 4 independent clones (WT; *53bp1*^*−/−*^), *n* = 3 (*Shld1*^*−/−*^), *n* = 2 (*Xlf*^*−/−*^; *Shld1*^*−/−*^ *Xlf*^*−/−*^; *53bp1*^*−/−*^ *Xlf*^*−/−*^), two-sided Wilcoxon–Mann–Whitney test (****p < 0.0001). WT: clones #O38 (*n* = 4 independent experiments), #GBB (*n* = 4), #12096 (*n* = 4) and #12095 (*n* = 11); *53bp1*^*−/−*^: clones #9999 (n = 7), #1110 (n = 3), #BP95-2 (n = 3) and #BP95-5 (n = 3); *Shld1*^*−/−*^: clones #O32 (n = 3), #O37 (*n* = 6) and #O44 (*n* = 3); *Xlf*^*−/−*^: clones #16488 (*n* = 10) and #X95-3 (*n* = 2); *Shld1*^*−/−*^*Xlf*^*−/−*^: clones #XO2-8 (*n* = 4) and #XO2-24 (*n* = 6); *Xlf*^*−/−*^*53bp1*^*−/−*^: clones #9X1 (*n* = 8) and #9X2 (*n* = 6). n.s. non-significant (*p* ≥ 0.05), *p < 0.05, ****p < 0.0001. Source data are provided as a Source Data file.

Consistent with the analysis of lymphocyte progenitor populations, semi-quantitative PCR analysis of Dβ1 to Jβ1 and Dβ2 to Jβ2 rearrangements in thymocytes from WT, *Shld1*^*−/−*^, *Xlf*^*−/−*^, and *Shld1*^*−/−*^ *Xlf*^*−/−*^ mice showed similar levels of rearrangements (Fig. 2a–c and Supplementary Fig. 2a, b). Notably, Dβ1 to Jβ1 and Dβ2 to Jβ2 rearrangements were also robustly detected in *53bp1*^*−/−*^ mice (Fig. 2a–c and Supplementary Fig. 2a, b). We next tested whether SHLD1, similar to 53BP1^18^, might facilitate the joining of more distant V(D)J gene segments. We performed quantitative PCR assays of partial (Dδ2-Jδ1 and Dδ1-Dδ2) and complete (Vδ5-Dδ1 and Vδ2-DδJδ1) rearrangements at the *Tcrδ* locus (Fig. 2d, e). Consistent with previous findings, we found that short-range (Dδ2-Jδ1 and Dδ1-Dδ2) rearrangements were similar to or even more abundant in *53bp1*^*−/−*^ than in WT thymocytes (Fig. 2d, e). In contrast, long-range Vδ-to-DδJδ recombination was significantly reduced in *53bp1*^*−/−*^ thymocytes (Fig. 2d, e). Strikingly, *Shld1*^*−/−*^ thymocytes showed similar levels of rearrangements as WT thymocytes (Fig. 2d, e), demonstrating that SHLD1 is dispensable for long-range V(D)J recombination.

To more robustly quantify V(D)J recombination, we generated viral-Abelson kinase (*v-Abl*) transformed pro-B cell lines from wildtype (WT) and *Shld1*^*−/−*^ mice^40^. Treatment of *v-Abl* transformed pro-B cells with a *v-Abl* kinase inhibitor (hereafter named ABLki) leads to G1 cell cycle arrest, the rapid induction of RAG1/2 gene expression, and rearrangement of the *Igk* locus or introduced V(D)J recombination reporter substrates^40^. PCR analysis of *IgkV*_*6–23*_*-J*_*1*_ rearrangements revealed normal levels of recombination in *Shld1*^*−/−*^ pro-B cells (Fig. 2f and Supplementary Fig. 2c). In addition, quantitative flow cytometry analysis of *v-Abl* pro-B cells carrying the pMX-RSS-GFP/IRES-hCD4 retroviral recombination substrate (pMX-INV) that allows for GFP expression upon successful chromosomal inversional RAG-mediated recombination revealed similar levels of recombination in WT and *Shld1*^*−/−*^ *v-Abl* cells (Fig. 2h and Supplementary Fig. 2d, e). We next employed CRISPR/Cas9-mediated gene editing to delete *Shld1* from WT and *Xlf*^*−/−*^ *v-Abl* pro-B cells^38,41^, generating *Shld1*^*−/−*^ and *Shld1*^*−/−*^ *Xlf*^*−/−*^ pro-B cell clones. In addition, we deleted *Xlf* from *53bp1*^*−/−*^ *v-Abl* pro-B cells^21^, generating *Xlf*^*−/−*^ *53bp1*^*−/−*^ pro-B cell clones. Consistent with the previous work^20,21,38,41^, both PCR analysis of *Igk* rearrangements and flow cytometry analysis of pMX-INV recombination revealed robust recombination in XLF-deficient pro-B cells and a severe defect in V(D)J rearrangements in 53BP1/XLF-doubly deficient pro-B cells (Fig. 2f–i and Supplementary Fig. 2e). Strikingly, in contrast to 53BP1/XLF-deficient pro-B cells, SHLD1/XLF-deficient pro-B cells perform robust V(D)J recombination at the *Igk* locus as well as at the pMX-INV substrate (Fig. 2f–i and Supplementary Fig. 2e). Together, these results demonstrate that SHLD1 is dispensable for V(D)J recombination even in the absence of XLF and that 53BP1 has unique functions during V(D)J recombination that are independent of SHLD1.

### SHLD1 is required for class switch recombination

**Fig. 3:**
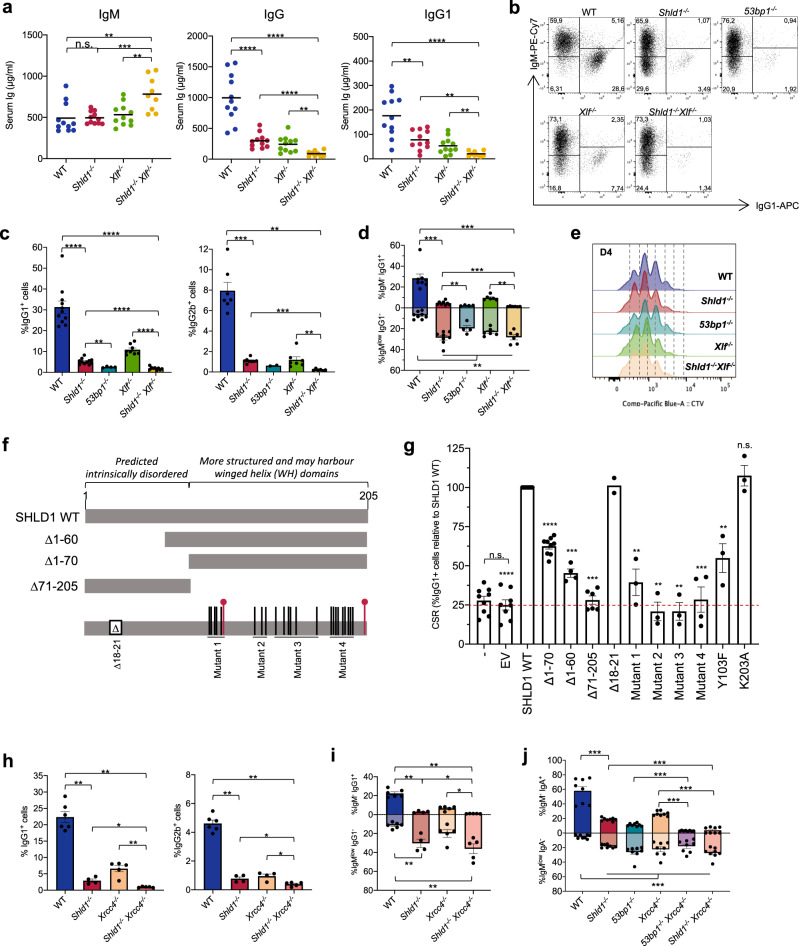
SHLD1 is required for class switch recombination. **a** Concentration of indicated isotypes in the serum of 6‐ to 8‐week‐old sex- and aged-matched unimmunized WT, *Shld1*^*−/−*^*, Xlf*^*−/−*^, *and Shld1*^*−/−*^*Xlf*^*−/−*^ mice. Horizontal lines show mean, n = 11 independent mice (except *Shld1*^*−/−*^ *Xlf*^*−/−*^ for which *n* = 9), two-sided Wilcoxon–Mann–Whitney test, *p* values are indicated in Source Data file. **b** Representative dot plots showing IgM and IgG1 expression in WT, *Shld1*^*−/−*^*, 53bp1*^*−/−*^*, Xlf*^*−/−*^, and *Shld1*^*−/−*^*Xlf*^*−/−*^ stimulated B cells. **c** Percentages of WT, *Shld1*^*−/−*^*, 53bp1*^*−/−*^*, Xlf*^*−/−*^*,* and *Shld1*^*−/−*^*Xlf*^*−/−*^ B cells expressing either IgG1 or IgG2b isotype after 4 days of in vitro stimulation with anti-IgD dextran, LPS and IL-4 (IgG1) or LPS (IgG2b). Bars represent mean ± s.e.m., WT (*n* = 11 for IgG1 and *n* = 7 for IgG2b), *Shld1*^*−/−*^ (*n* = 11 for IgG1 and *n* = 8 for IgG2b), *53bp1*^*−/−*^ (*n* = 4 for IgG1 and *n* = 2 for IgG2b), *Xlf*^*−/−*^ (*n* = 8 for IgG1 and *n* = 7 for IgGb2), *Shld1*^*−/−*^ *Xlf*^*−/−*^ (*n* = 9 for IgG1 and *n* = 6 for IgG2b), two-sided Wilcoxon–Mann–Whitney test, *p* values are indicated in Source Data file. **d** Percentages of IgM^−^IgG1^+^ and IgM^low^ IgG1^−^ cells. Bars represent mean ± s.e.m., *n* = 8 (except *53bp1*^*−/−*^ for which *n* = 4, and *Xlf*^*−/−*^ and Shld1^−/−^ *Xlf*^*−/−*^ for which *n* = 6), two-sided Wilcoxon–Mann–Whitney test, *p* values are indicated in Source Data file. **e** CTV dilution assay in purified B cells cultured in the presence of anti-IgD dextran, LPS, and IL-4 for 96 h. Representative data, *n* > 2 mice. **f** SHLD1 predicted domains and variants used. Mutant 1: L95A, R96A, S98A, L99A, F102A, Y103A; Mutant 2: L125A, I129A, L132A; Mutant 3: Y140A, M147A, V150A, I151A, D155A, F169A; Mutant 4: P186A, G187A, L188A, S189A, D191A, I192A, F195A, L196A, L197A. **g** CSR assay in *Shld1*^*−/−*^ primary B cells complemented with SHLD1 variants. Bars represent the average ± s.e.m. relative to *Shld1*^*−/−*^ primary B cells complemented with SHLD1 WT, *n* = 9 independent experiments (non-complemented cells and Δ1–70), *n* = 8 (EV), *n* = 10 (SHLD1 WT), *n* = 4 (Δ1–60 and Mutant 4), *n* = 6 (Δ 71–205), *n* = 2 (Δ18–21), *n* = 3 (Mutant 1; Mutant 2; Mutant 3; Y103F; K203A), two-sided Wilcoxon–Mann–Whitney test (****p < 0.0001 (vs EV; vs Δ1–70); ***p = 0.001 (vs Δ1–60; vs Mutant 4); ***p = 0.0001 (vs Δ71–205); **p = 0.0035 (vs Mutant 1; vs Mutant 2; vs Mutant 3; vs Y103F)). **h** Percentages of WT, *Shld1*^*−/−*^, *Xrcc4*^*−/−*^ and *Shld1*^*−/−*^*Xrcc4*^*−/−*^ B cells expressing either IgG1 or IgG2b isotype after 4 days of in vitro stimulation with anti-IgD dextran, LPS and IL-4 (IgG1) or LPS (IgG2b). Bars represent mean ± s.e.m., WT (*n* = 6), *Shld1*^*−/−*^ (*n* = 4), *Xrcc4*^*−/−*^ (*n* = 5 for IgG1 and *n* = 4 for IgG2b) and *Shld1*^*−/−*^*Xrcc4*^*−/−*^(*n* = 5), two-sided Wilcoxon–Mann–Whitney test, *p* values are indicated in Source Data file. **i** Percentages of IgM^**−**^IgG1^+^ versus IgM^low^ IgG1^−^ cells. Bars represent mean ± s.e.m., *n* = 6 (WT), *n* = 4 (*Shld1*^*−/−*^), *n* = 5 (*Xrcc4*^*−/−;*^ *Shld1*^*−/−*^*Xrcc4*^*−/−*^), two-sided Wilcoxon–Mann–Whitney test, *p* values are indicated in Source Data file. **j** Percentages of IgM^−^ IgA^+^ versus IgM^low^ IgA^−^ CH12 cells after in vitro stimulation with anti-CD40 antibody, IL-4, and TGF-β for the indicated genotypes. Bars represent mean ± s.e.m., *n* = 4 independent experiments with two clones per genotype, two-sided Wilcoxon–Mann–Whitney test, *p* values are indicated in Source Data file. n.s. non-significant (*p* ≥ 0.05), *p < 0.05, **p < 0.01, ***p < 0.001, ****p < 0.0001. Source data are provided as a Source Data file.

To determine the function of SHLD1 in CSR in vivo, we first measured the serum immunoglobulin levels in unimmunized mice (Fig. 3a). *Shld1*^*−/−*^ mice showed normal amounts of serum IgMs but reduced levels of total IgG and IgG1 antibodies, which is suggestive of defective CSR (Fig. 3a). To further test the role of SHLD1 in CSR, we purified splenic B cells and induced them to switch to IgG1, IgG2b, and IgG3 (Fig. 3b, c and Supplementary Fig. 3a–c). We found that SHLD1 deficiency severely compromised the production of IgG1-, IgG2b- and IgG3b-switched B splenocytes to approximately 15% of WT (Fig. 3b, c and Supplementary Fig. 3a–c). CSR defect in SHLD1-deficient B cells undergoing IgM-to-IgG1 switching was also associated with a large population of IgM^low^ IgG1^−^ B cells, suggesting aberrant AID-DSB repair^27^ (Fig. 3b, d). Finally, SHLD1 deficiency did not affect cell proliferation (Fig. 3e and Supplementary Fig. 3d), *Aid* expression and *Igh* germline transcripts levels (Supplementary Fig. 3e, f). These results indicate that SHLD1 is critical for CSR in mice, supporting our previous findings in CH12 B cell lines^32^. Notably, *53bp1*^*−/−*^ B cells harbored stronger defects in CSR compared to *Shld1*^*−/−*^ B cells (Fig. 3b, c and Supplementary Fig. 3a), suggesting that 53BP1 also fulfills SHLD1-independent functions during CSR.

To investigate further the function of SHLD1, we have tested a series of deletion and substitution mutations for their ability to rescue CSR in stimulated *Shld1*^*−/−*^ primary B cells (Fig. 3f). SHLD1 is encoded by two exons, exon 2 that encodes for amino acids 1 to 60 and exon 3 that encodes for amino acids 61 to 205. The SHLD1 N-terminus (1–70) is predicted to be intrinsically disordered while its C-terminal part is more structured and may harbor one- or two-winged helix domains^32^. To investigate the function of the N- and C-terminal regions of SHLD1, we tested WT SHLD1 as well as SHLD1^Δ1–60^, SHLD1^Δ1–70^, and SHLD1^Δ71–205^ deletion mutants (Fig. 3f). In addition, we generated a series of deletion and combined substitution mutations at strongly conserved residues (SHLD1^Δ18-21^ and SHLD1^L95A, R96A, S98A, L99A, F102A, Y103^, SHLD1^L125A, I129A, L132A^, SHDL1^Y140A, M147A, V150A, I151A, D155A, F169A^, and SHLD1^P186A, G187A, L188A, S189A, D191A, I192A, F195A, L196A, L197A^ referred to as mutants 1, 2, 3, and 4, respectively (Fig. 3f, and Supplementary Fig. 4a). We found that SHLD1^Δ1-60^ and SHLD1^Δ1-70^ partially restored CSR in stimulated *Shld1*^*−/−*^ cells while SHLD1^Δ71-205^ and any of the tested SHLD1 proteins mutated for highly conserved residues in the C terminus failed to rescue CSR in stimulated SHLD1-deficient B cells (Fig. 3g). Of note, we could not detect SHLD1^Δ71-205^ by Western Blot suggesting that this mutant form of SHLD1 is unstable (Supplementary Fig. 4b). In contrast, SHLD1^Δ18-21^ restored CSR to levels equivalent to those measured after complementation with WT SHLD1 indicating that these four amino acid residues, recently implicated in CTC1-STN1-TEN1 (CST)-Polα-primase binding^42^, are dispensable for CSR (Fig. 3g). Together, these results show that the C-terminal (i.e., amino acids 71 to 205) portion of SHLD1 forms the core region of SHLD1 that is required for CSR. Finally, we generated substitution mutations at residues Y103 (SHLD1^Y103F^) and K203 (SHLD1^K203A^) that are invariably conserved in diverse species and represent potential phosphorylation and ubiquitylation sites, respectively^43^. We found that SHLD1^K203A^ fully rescued CSR in SHLD1-deficient B cells. By contrast, SHLD1^Y103F^ only partially restored IgG1 class switching suggesting that this residue might be implicated in SHLD1 function during CSR. SHLD1^Y103F^ and SHLD1^Δ1-70^ were still able to interact with REV7/MAD2L2 (Supplementary Fig. 4b), suggesting that the Y103 residue and the SHLD1 N-terminus are dispensable for SHLD1 binding to upstream SHLD complex components.

### 53BP1-SHLD and NHEJ synergize during class switch recombination

We next investigated potential synergistic or epistatic functions between SHLD1 and XLF during CSR. Strikingly, *Shld1*^*−/−*^ *Xlf*^*−/−*^ mice displayed a significant increase of serum IgMs as compared to wildtype and single mutant mice that was associated with an almost complete lack of total IgG and IgG1 antibodies, indicating that CSR is highly defective in SHLD1/XLF-deficient activated B cells (Fig. 3a). Consistent with this, class switching to IgG1, IgG2b and IgG3 was almost completely abrogated in SHLD1/XLF-deficient stimulated splenic B cells (Fig. 3b, c and Supplementary Fig. 3a, b) without noticeable defects in cell proliferation, *Aid* expression, and *Igh* germline transcripts levels (Fig. 3e and Supplementary Fig. 3d–f). In addition, defective CSR in *Shld1*^*−/−*^, *Xlf*^*−/−*^, and *Shld1*^*−/−*^ *Xlf*^*−/−*^ splenic B cells induced to switch to IgG1 was associated with a large population of IgM^low^ IgG1^−^ B cells that inversely correlated with the levels of switched IgG1^+^ cells in the respective mutant cells (Fig. 3b, d). Together, these results demonstrate that SHLD1 and XLF act synergistically to promote productive CSR.

To probe the capacity of SHLD1 to promote CSR in the absence of XRCC4, another NHEJ factor, we generated SHLD1-proficient and SHLD1-deficient mice that harbored two copies of a loxP-flanked (floxed) Xrcc4 allele (*Xrcc4*^*f*^)^44^ plus a transgene that drives Cre recombinase expression in late stages of the B lineage from a *CD21* promoter^45^, termed *CD21-cre*^*Tg*^ *Xrcc4*^*f/f*^ mice and *Shld1*^*−/−*^ *CD21-cre*^*Tg*^ *Xrcc4*^*f/f*^ mice respectively. Consistent with previous work^46^, flow cytometry assays for surface IgG1 and IgG2b revealed substantial switching by appropriately stimulated *CD21-cre*^*Tg*^*Xrcc4*^*f/f*^ B cells with average levels of IgG1 and IgG2b that were at about 25% of those of WT controls (Fig. 3h). Similar to SHLD1/XLF-deficient B cells, class switching to IgG1 or IgG2b was significantly diminished in SHLD1/XRCC4-deficient stimulated B cells and was associated with a large population of IgM^low^ IgG1^−^ B cells (Fig. 3h, i). Thus, SHLD1 supports CSR in the absence of XRCC4.

We next investigated the capacity of 53BP1 to promote CSR in the absence of XRCC4. We used CRISPR/Cas9 gene editing to delete *Xrcc4* from wildtype, *Shld1* knockout, and *53bp1* knockout CH12F3 (CH12) B cell clones, generating *Xrcc4*^*−/−*^, *Shld1*^*−/−*^ *Xrcc4*^*−/−*^, and *53bp1*^*−/−*^ *Xrcc4*^*−/−*^ B cell clones, respectively. We established that combined loss of SHLD1 and XRCC4 or 53BP1 and XRCC4 almost completely abrogates the production of IgA-switched CH12 cells as measured by flow cytometry after stimulation with anti-CD40, IL-4, and TGF-β (Fig. 3j). Together, these results indicate that 53BP1-SHLD is essential for “productive” CSR—that is the ability of switched cells to express a new immunoglobulin isotype at the cell surface – in both NHEJ-proficient and NHEJ-deficient B cells.

### SHLD1 promotes productive CSR in XLF-proficient and XLF-deficient cells by limiting DNA end resection

**Fig. 4:**
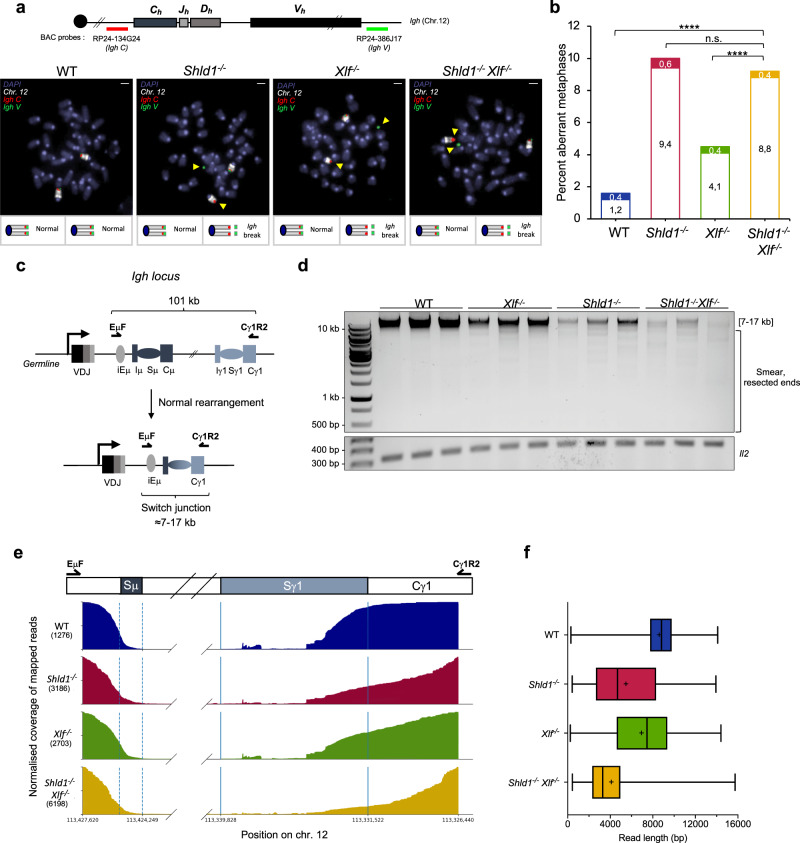
SHLD1 promotes functional CSR in XLF-proficient and XLF-deficient cells by limiting DNA end resection. **a** Representative images of *Igh* breaks in aberrant metaphases, as quantified in b. Scale bars, 3 μm. **b** Quantification of *Igh* breaks (clear bar) and translocations (filled bar) in metaphases of WT, *Shld1*^*−/−*^*, Xlf*^*−/−*^, and *Shld1*^*−/−*^*Xlf*^*−/−*^ cells. Bars represent means. *n* = 1005 (WT), *n* = 1075 (*Shld1*^*−/−*^*)*, *n* = 1201 (*Xlf*^−/−^), *n* = 1000 (*Shld1*^*−/−*^ *Xlf*^*−/−*^), two-sided Fisher’s exact test from four independent experiments (****p < 0.0001). See also Supplementary Table 4. **c** Schematic representation of the murine *Igh* locus. Arrows indicate primers used for the long-range PCR. **d** Long-range PCR analysis of Sμ to Sγ1 rearrangements in WT, *Xlf*^*−/−*^, *Shld1*^*−/−*^, and *Shld1*^*−/−*^*Xlf*^*−/−*^ stimulated B cells. *Il2* gene PCR was used as a loading control. **e** Normalized coverage of the mapped PacBio reads in WT, *Shld1*^*−/−*^, *Xlf*^*−/−*^, and *Shld1*^*−/−*^ *Xlf*^*−/−*^ stimulated B cells. Vertical dashed lines indicate the Sμ switch region and vertical lines indicate the Sγ1 region. **f** Distribution of read length (in bp) in WT, *Shld1*^*−/−*^, *Xlf*^*−/−*^, and *Shld1*^*−/−*^ *Xlf*^*−/−*^ cells. *n* = 3 independent samples. For all boxplots: minima is minimum value, maxima is maximum value, center is median and quartiles shown by box and whiskers, and cross is mean. n.s. non-significant (*p* ≥ 0.05), ****p < 0.0001. Source data are provided as a Source Data file.

The most severe CSR defect of B cells deficient for SHLD1 and XLF could be due to a lack of repair of AID-induced DSBs or, as another non-mutually exclusive possibility, to hyper-resection leading to deletions of coding or regulatory regions essential for the expression of a functional switched immunoglobulin. To distinguish between these possibilities, we first measured the levels of *Igh*-DSBs by performing *Igh* locus-specific DNA–FISH on chromosome spreads prepared from wildtype, *Shld1*^*−/−*^, *Xlf*^*−/−*^, and *Shld1*^*−/−*^ *Xlf*^*−/−*^ splenic B cells stimulated to undergo IgM-to-IgG1 class switching (Fig. 4a). Analysis of metaphase spreads revealed low levels of *Igh* locus-associated chromosomal breaks in WT B cells (1.2%). *Xlf*^*−/−*^ and *Shld1*^*−/−*^ B cells contained a statistically significant increase in aberrant metaphases (4.1% and 9.4% respectively) that could reflect either a slower repair or a partial lack of repair of AID-induced DSBs in these cells (Fig. 4a, b and Supplementary Table 4). Interestingly, despite an almost complete absence of IgG1 switched cells 4 days after stimulation, *Xlf*^*−/−*^ *Shld1*^*−/−*^ B cells contained similar levels of *Igh*-associated chromosomal breaks (8.8%) as SHLD1 single-deficient cells (Fig. 4b). Similarly, metaphase spreads from *Shld1*^*−/−*^ *CD21-cre*^*Tg*^ *Xrcc4*^*f/f*^ stimulated B cells contained approximately the same level of *Igh*-associated chromosomal breaks as metaphases from *Shld1*^*−/−*^ B cells (Supplementary Fig. 5a, b and Supplementary Table 5). These results indicate that, in the absence of both NHEJ (*i.e*. XLF or XRCC4) and SHLD1, AID-induced DSBs might be aberrantly resolved, leading to the generation of unproductive switched-joins, rather than accumulating in the form of unrepaired DNA breaks.

To analyze AID-mediated recombining products, we extracted genomic DNA from stimulated splenic B cells and adapted a long-range PCR assay to amplify a region between the *Igh* intronic enhancer iEμ (primer EμF) and the *Igh γ1* constant exon 6 (primer Cγ1R2) (Fig. 4c). The EμF and Cγ1R2 primers locate more than 100 kb apart and therefore do not allow amplification of the genomic sequence in the germline configuration or after AID-mediated inversional recombination between the *Sμ* region and the *Sγ1* region. Amplification of genomic DNA from stimulated WT splenic B cells generated a clear band whose size corresponds to the expected deletional recombination products between the *Sμ* region and the *Sγ1* region (i.e., from 7160 bp to 16,760 bp product sizes) (Fig. 4d). Amplification of genomic DNA from *Xlf*^*−/−*^ and *Shld1*^*−/−*^ B cells generated a weaker band of the expected recombination size that is consistent with the reduced percentage of IgG1^+^ cells in these cells and, instead, produced smaller PCR fragments that might correspond to resection of DNA ends prior to joining (Fig. 4d). The proportion of small PCR fragments was quite similar in SHLD1 knockout samples and 53BP1 knockout samples and greater than in XLF knockout samples (Fig. 4d and Supplementary Fig. 5c). Most strikingly, *Shld1*^*−/−*^ *Xlf*^*−/−*^ B cells harbored recombination products of various sizes that could be visualized as a smear on the agarose gel and that suggest intense resection of AID-induced DSBs in these cells (Fig. 4d and Supplementary Fig. 5c). To confirm these results, we sequenced PCR products obtained from wildtype and mutant B cells using long-read high-throughput sequencing to analyze hundreds of individual sequences from each genotype (Fig. 4e). This analysis revealed that in WT B cells the vast majority of the sequences corresponded to recombination products with 5′ and 3′ breakpoint sites mapping within the *Sμ* core region and the *Sγ1* core region (Fig. 4e) with a mean read length of 8625 bp and with 15.6% of the reads being smaller than 7160 bp, which correspond to recombination products that suffered intense resection of DNA ends prior to joining (Fig. 4e, f and Supplementary Table 6). XLF-deficiency led to the generation of smaller recombination products (mean read length = 7035 bp) with 46.8% of the reads smaller than 7160 bp (Fig. 4e. f and Supplementary Table 6). Most strikingly, SHLD1-deficient B cells contained switched products with 66.4% of the reads smaller than 7160 bp in *Shld1*^*−/−*^ B cells (mean length = 5478 bp) and 85.3% of the reads smaller than 7160 bp in *Shld1*^*−/−*^ *Xlf*^*−/−*^ B cells (mean length = 4175 bp) (Fig. 4e, f and Supplementary Table 6). Thus, SHLD1 promotes productive CSR in XLF-proficient and XLF-deficient B cells by antagonizing and controlling the extent of DNA end resection.

ATM plays a dual role in CSR by promoting 53BP1-RIF1-SHLD assembly at AID-induced DSBs and participating in CtIP-mediated nuclease activities that contribute to DNA end resection. To test the impact of ATM ablation in the context of SHLD1 deficiency, we bred our *Shld1*^*−/−*^ mice with *Atm*^*−/−*^ mice^38,47^ to generate doubly deficient animals. *Shld1*^*−/−*^ *Atm*^*−/−*^ mice were born at expected frequencies and displayed no additional deleterious developmental or immune phenotype compared with *Atm*^*−/−*^ mice (Supplementary Fig. 6a–d), indicating that *Atm* and *Shld1* are epistatic during development and for immune functions such as V(D)J recombination. Analysis of class switching to IgG1 and IgG2b in stimulated naive B cells revealed similar levels of class switching in *Shld1*^*−/−*^ *Atm*^*−/−*^ cells as compared to *Atm*^*−/−*^ and *Shld1*^*−/−*^ cells (Supplementary Fig. 6e). Although not statistically significant, we noticed a small increase in the percentage of switched cells in *Shld1*^*−/−*^ *Atm*^*−/−*^ as compared to *Shld1*^*−/−*^ cells, suggesting that ATM deficiency might improve the production of functional joins in SHLD1-deficient B cells rather than aggravate it, possibly by dampening DNA end resection in these cells. Long-range PCR analysis of recombining products recovered from stimulated *Shld1*^*−/−*^ *Atm*^*−/−*^ splenic B cells showed that the loss of ATM in SHLD1-deficient cells partially restored high molecular weight PCR products in SHLD1-deficient B cells (Supplementary Fig. 6f), indicating that ATM might promote end resection in SHLD1-deficient B cells. Consistent with this, inhibition of the ATM kinase activity in stimulated SHLD1-deficient splenic B cells substantially restored CSR levels and high molecular weight recombination products (Supplementary Fig. 6g, h).

### SHLD1 deficiency and long DNA end resection leads to unbiased directional joining during CSR

**Fig. 5:**
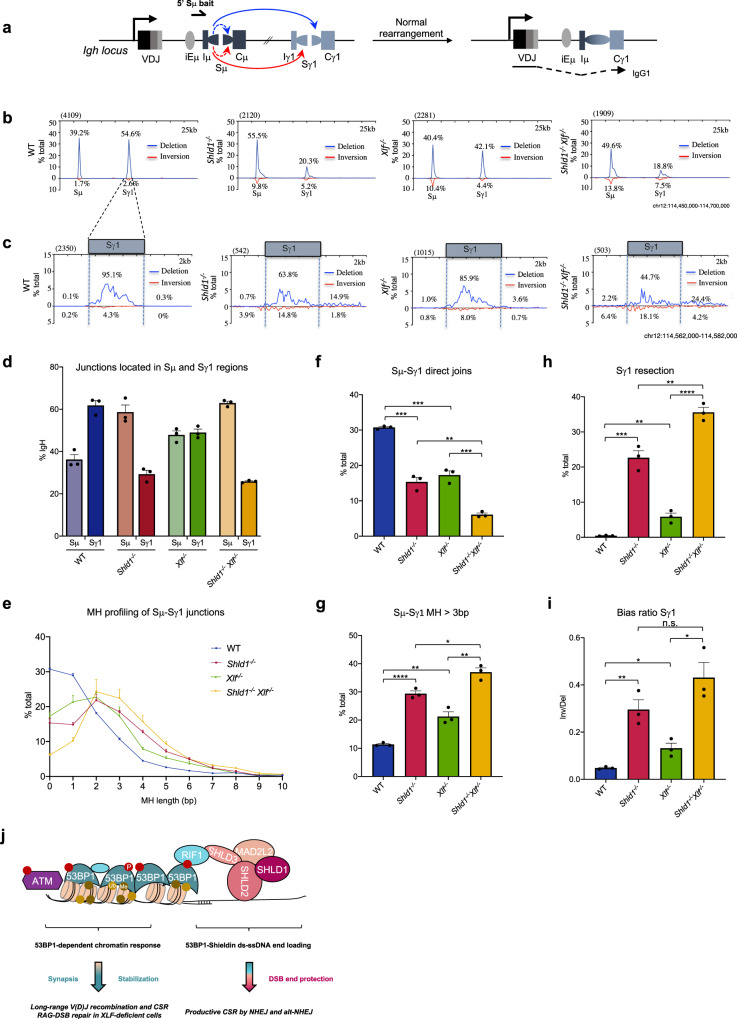
SHLD1 deficiency and long DNA end resection leads to unbiased directional joining during CSR. **a** Illustration of activation of CSR in normal B cells stimulated with anti-IgD dextran/LPS/IL-4 which induces AID and activates transcription of Iγ1. Deletional CSR events are indicated with a blue arrow and inversional recombination events are indicated with a red arrow. Intra-switch recombination events are indicated with a dotted line. **b** CSR-HTGTS-seq analysis of break joining between 5′Sμ and downstream acceptor S-regions in WT, *Shld1*^*−/−*^, *Xlf*^*−/−*^, and *Shld1*^*−/−*^*Xlf*^*−/−*^ splenic B cells stimulated with anti-IgD dextran/LPS/IL-4. The blue line indicates deletional joining, and the red line indicates inversional joining. **c** Zoom-in view of CSR-HTGTS-Seq junctions located in the AID-targeted ectopic Sγ1 region from WT, *Shld1*^*−/−*^, *Xlf*^*−/−*^, and *Shld1*^*−/−*^*Xlf*^*−/−*^ cells. Junctions are plotted at 200 bp bin size. **d** Bar graph showing percentages of junctions located in Sμ and Sγ1 regions from WT, *Shld1*^*−/−*^, *Xlf*^*−/−*^, and *Shld1*^*−/−*^*Xlf*^*−/−*^ splenic B cells stimulated with anti-IgD dextran/LPS/IL-4. Data represent mean ± s.e.m. from three independent repeats. **e** Microhomologies (MH) usage of Sμ-Sγ1 junctions, plotted as a percentage of total junctions. *n* = 3 independent samples. **f**, **g** Percentages of Sμ-Sγ1 direct joins in **f** and Sμ-Sγ1 joins with 4-bp or longer MH in **g** in the different genetic backgrounds are compared. Bars represent mean ± s.e.m. *n* = 3 independent samples, unpaired two-tailed *t*-test, *p* values are indicated in Source Data file. **h** Percentage of resection at the Sγ1 region in the different genetic backgrounds as indicated. Bars represent mean ± s.e.m., *n* = 3 independent samples, unpaired two-tailed *t*-test, *p* values are indicated in Source Data file. **i** Inversional/deletional joining ratio at the Sγ1 region in the different genetic background as indicated. Bars represent mean ± s.e.m., *n* = 3 independent samples, unpaired two-tailed *t*-test, *p* values are indicated in Source Data file. n.s. non-significant (*p* ≥ 0.05), *p < 0.05, **p < 0.01, ***p < 0.001, ****p < 0.0001. Source data are provided as a Source Data file. **j** Working model for the specific roles of 53BP1 and components of the SHLD complex in antigen receptor gene diversification. The 53BP1-dependent chromatin response acts independently of SHLD to promote chromosome structural changes that are essential for long-range V(D)J recombination and CSR as well as for RAG-DSB repair in the context of an unstable post-cleavage complex (i.e., XLF-deficient cells) (left panel), while the 53BP1-SHLD axis limits resection at AID-induced DSB ends, providing an end-protection mechanism that permits productive CSR by NHEJ and alt-NHEJ (right panel).

CSR is programmed to occur in a productive deletional orientation through a Cohesin-mediated loop extrusion mechanism that juxtaposes AID-initiated DNA ends within donor and acceptor S-regions for deletional CSR^14,48^. Deletional repair of AID-induced DSBs at the *Igh* locus is dependent on 53BP1 as residual junctions from 53BP1-deficient cells display a more normalized ratio of deletional versus inversional end-joining^14^. To test the potential role of SHLD1 in orientation-specific CSR, we used high-throughput genome-wide translocation sequencing (HTGTS), a linear amplification-based method that identifies “prey” DSB junctions to a fixed “bait” DSB with nucleotide resolution to simultaneously quantify end resection, microhomology (MH) usage and orientation of AID-generated DNA end-joining between the 5′ region of Sμ (the bait) and the downstream core Sγ1 region (the prey)^13,14,48^ (Fig. 5a). For this study, we analyzed >5,000 junctions from three independent mice of each wildtype, *Shld1*^*−/−*^, *Xlf*^*−/−*^, and *Shld1*^*−/−*^ *Xlf*^*−/−*^ genotype. Results from these experiments were highly reproducible (Fig. 5b, c and Supplementary Fig. 7).

Consistent with class switching levels in these cells, XLF-deficient B cells, SHLD1-deficient B cells, and SHLD1/XLF-deficient B cells had reduced Sγ1 junctions compared to wild-type B cells with *Shld1*^*−/−*^ *Xlf*^*−/−*^ cells having the most dramatic decrease in Sμ-Sγ1 joins (Fig. 5b–d). In WT cells, breakpoint junctions are almost exclusively located in S-regions with a very small fraction of the Sγ1 breakpoint sites being outside the core Sγ1 region (0.45%) and corresponding to long resection^14^ (Fig. 5c). Junctions were approximately 30% direct, with most of the others using 1 bp to 3 bp of MH and a small fraction (11%) using longer MHs (Fig. 5e–g). In agreement with previous studies in NHEJ-deficient cells^13,49,50^, XLF-deficient B cells had a marked increase in MH-mediated Sμ–Sγ1 junctions, with about 17% being direct and a correspondingly increased fraction of MH-mediated junctions (MH >3 bp = 21%), indicating increased use of alternative end-joining in these cells^9,10^. In addition, in XLF-deficient B cells, a small but significantly increased fraction of Sμ–Sγ1 junctions harbored long resection (6%). Consistent with results from long-range PCR, SHLD1-deficient B cells had greater long resection increases (23%) and SHLD1/XLF-deficient B cells far greater increases (36%) that were also apparent as a ‘flattening’ of Sγ1 junction profile relative to other backgrounds (Fig. 5c, h). Notably, MHs were also significantly more present and longer at junctions recovered from SHLD1-deficient and, to a greater extent, SHLD1/XLF-deficient B cells (*Shld1*^*−/−*^ cells: direct joints = 15%, MH >3 bp = 29%; *Shld1*^*−/−*^ *Xlf*^*−/−*^ cells: direct joints = 6%, MH >3 bp = 37%) as compared with XLF-deficient and wild-type B cells (Fig. 5e–g). Thus, SHLD1 restricts end resection and controls microhomology-mediated alternative end-joining of AID-initiated DSBs in both NHEJ-proficient and NHEJ-deficient B cells.

Remarkably, SHLD1-deficient B cells displayed a profound defect in orientation-specific joining (Fig. 5i), as observed in 53BP1-deficient cells^14,50^. Compared to wild-type B cells, where Sμ–Sγ1 recombination occurs predominantly by deletion (>95%), in *Shld1*^*−/−*^ B cells deletions and inversions were present at 70 and 30%, respectively (Fig. 5i). In *Shld1*^*−/−*^ *Xlf*^*−/−*^ B cells, deletions and inversions were present at nearly equal frequencies (57 vs 43%) (Fig. 5i). This normalization of the orientation ratios correlated with the level of DNA end resection in these two B cell types (Fig. 5h), suggesting that, in the absence of SHLD1, exacerbated resection hinders orientation-specific CSR. In support of this and in agreement with previous work in NHEJ-deficient cells^13,14,50^, XLF deficiency, which has a much less severe impact on CSR than SHLD1 deficiency and does not promote nearly the same degree of long S resections, also moderately impacted orientation-specific CSR (87 deletion vs 13% inversion). Altogether, these results suggest that the extent of AID-DSB end resection impacts orientation-specific CSR.

## Discussion

The SHLD complex acts downstream of 53BP1-RIF1 to counteract DSB end resection and promote NHEJ, a DSB repair pathway essential for V(D)J recombination and CSR. Here we find that SHLD1 is dispensable for lymphocyte development and V(D)J recombination, but does however play an essential role in ensuring productive CSR in B cells. We propose a model in which 53BP1 promotes antigen receptor gene diversification through two distinct mechanisms. First, the 53BP1-dependent chromatin DSB response acts independently of SHLD to promote chromosome structural changes that are essential for CSR, long-range V(D)J recombination, and RAG-DSB repair in the context of an unstable post-cleavage complex (i.e., XLF-deficient cells). Second, the 53BP1-SHLD axis limits resection at AID-induced DSB ends, providing an end-protection mechanism that permits productive CSR by NHEJ and alt-NHEJ (Fig. 5j).

Contrary to *53bp1*^*−/−*^ mice that display mild lymphopenia (^16–18,30^ and this study), we find that *Shld1*^*−/−*^ mice harbor normal numbers of B and T cell populations in lymphoid organs. *Shld2*^*−/−*^ mice^27^ and REV7-deficient animals^30^ also show normal lymphocyte differentiation, suggesting that all components of the SHLD complex are dispensable for lymphocyte differentiation and V(D)J recombination. These results imply that RAG-generated DSB ends do not require SHLD protection against resection prior to joining. Consistent with this, we find that *Shld1*^*−/−*^ *Xlf*^*−/−*^ mice support lymphocyte development and *Shld1*^*−/−*^ *Xlf*^*−/−*^ lymphocytes perform RAG-induced DSB repair at levels similar to those observed in *Xlf*^*−/−*^ lymphocytes. This is again in striking contrast to the severe combined immunodeficiency phenotype observed in *53bp1*^*−/−*^ *Xlf*^*−/−*^ animals and the accumulation of resected RAG-induced DSB ends observed in *53bp1*^*−/−*^ and *53bp1*^*−/−*^ *Xlf*^*−/−*^ progenitor lymphocytes^18,20,21^.

Why is the SHLD complex, contrary to 53BP1, dispensable for RAG-induced DSB repair? Specific features of the V(D)J recombination reaction might provide clues to this question^2,51^. First, the RAG-cleavage and repair steps of the reaction are coupled, possibly limiting fortuitous end resection events. Second, RAG-generated DSB end structures are prone to repair by NHEJ, which means that hairpin-sealed coding ends are open, processed, and repaired by NHEJ and blunt signal ends require minimal end-processing prior to ligation by LIG4. Third, RAG-cleavage occurs in a very strict G1 environment that offers limited end resection activities. Additionally, during V(D)J recombination, ATM and downstream chromatin DSB response factors such as H2AX and 53BP1 contribute to DSB end-tethering and synapsis, a function that is thought to be redundant with XLF^19^. Functional compensation for DSB end-tethering/synapsis in the absence of XLF may also reside in additional post-cleavage complex proteins including RAG^38^, MRI^52^, and ERCC6L2^50^. In addition, XLF-deficiency is largely compensated by PAXX during end-joining^41,53,54^. Thus, even in the absence of XLF, RAG-generated DNA ends benefit from robust end-joining as well as a chromatin environment that alleviates the need for SHLD to limit resection. Our finding that *Shld1*^*−/−*^ *Xlf*^*−/−*^ mice do not suffer developmental growth defects, as opposed to *53bp1*^*−/−*^ *Xlf*^*−/−*^ mice^20,21^, *Atm*^*−/−*^ *Xlf*^*−/−*^ mice^22^, or *H2ax*^*−/−*^ *Xlf*^*−/−*^ mice^22^, indicates that the ATM-H2AX-53BP1-mediated chromatin DSB response also supports important SHLD-independent functions during mammalian development.

Two distinct features of 53BP1 explain its essentiality for CSR; the ability to form stable oligomeric assemblies at DSB sites and the faculty to protect DNA ends against resection mediated by RIF1 and components of the SHLD complex^23,24^. It has been proposed that tetramerization (and higher-order assembly) of 53BP1 might provide a tethering activity to bridge and stabilize distally located DNA ends such as in the case of AID-induced DSBs in donor and acceptor switch regions and RAG-induced DSBs in distant V(D)J segments^23,24^. Notably, mutations that interfere with 53BP1 higher-order oligomer formation abrogate CSR without substantially affecting DSB end resection^55^, suggesting that this function is independent of downstream RIF1-SHLD factors. In agreement with this, we show that 53BP1 knockout splenic B cells have a CSR defect approximately twice as severe as SHLD1 knockout splenic B cells, possibly illustrating the dual property of 53BP1. In addition, we find that recombination of distant V(D)J segments is independent of SHLD1. It was recently reported that DSB end protection might in fact play a limited role in the ability of 53BP1 to support CSR, based on the observation that a mutant form of 53BP1 defective for RIF1 recruitment still supports robust CSR^55^. Yet, B cells deficient for any component of the SHLD complex as well as for RIF1 display defective CSR^27–34,56–58^. The recent identification of distinct modes of RIF1 and SHLD recruitment at DSBs and action to promote DNA repair might partially explain this discrepancy^59^.

We find that murine *Shld1*^*−/−*^ B cells are defective for CSR due to unbiased orientation-joining of AID-DSBs and extensive DSB end resection in these cells. Inversional recombination leads to non-productive CSR as constant region coding sequences downstream of the donor Sμ region would be inverted and incapable of being translated^14^. Overactive resection also leads to non-productive CSR due to the loss of coding exons in the downstream acceptor constant region^27^.

Orientation-specific CSR is known to be dependent on 53BP1^14^ which could reflect the two aforementioned aspects of 53BP1: (1) a specialized structural role in promoting synapsis and stabilization of S-regions and/or (2) the ability to limit DNA end resection and promote rapid DNA repair, thus preventing the lingering of S-region broken ends within resection complexes and the loss of their orientation-specific joining properties. Interestingly, treatment of 53BP1 knockout activated B cells with an ATM kinase inhibitor substantially diminishes long S-region resections but does not restore orientation-dependent joining^14^, illustrating the intrinsic capacity of 53BP1 to promote orientation-specific CSR, possibly through stabilization of synapsed S-regions. However, as SHLD1 exerts its function at the very tip of the 53BP1-RIF1-SHLD axis when loaded onto ssDNA with SHLD2^32^, it is unlikely that its loss, per se, affects 53BP1 function in stabilizing DSBs. In support of this, SHLD1 loss does not perturb the accumulation of 53BP1 and RIF1 at DNA damage sites^28,32^. In addition, silencing of SHLD2 or SHLD3 does not affect the topological stabilization of DSB-flanking chromatin initiated by the formation of 53BP1 nano-domains^60^. Finally, we show that SHLD1 does not promote long-range V(D)J recombination, that is thought to rely on the ability of 53BP1 to facilitate chromosome synapsis^18^. Thus, we suggest that the loss of orientation-specific AID-DSB end-joining in SHLD1 knockout B cells is a result of excessive DNA end resection rather than a direct effect on the 53BP1-dependent chromatin DSB response. As Cohesin-mediated loop extrusion has been identified as a major mechanism enforcing deletional rearrangements during CSR^48,61^, one possible mechanistic explanation comes from the observation that although Cohesin has comparable affinities for single-stranded (ss) and double-stranded (ds) DNA, ssDNA binding is labile and topologically entrapped ssDNA can easily be lost from the Cohesin ring^62^. In addition, RPA can compete with Cohesin for ssDNA capture^62^ and thus could theoretically perturb Cohesin functions during DNA recombination. Thus, we speculate that in the absence of SHLD protection, very long stretches of ssDNA and/or RPA-coated ssDNA might interfere with Cohesin-driven loop extrusion of activated S-regions, leading to the defective orientation-specific joining of AID-induced DSBs. In support of this idea, we find that the extent of DNA end resection measured in XLF-, SHLD1- and SHLD1/XLF-deficient B cells correlates with the levels of inversional recombination in these cells.

SHLD acts in a paradoxical manner to hinder DNA end resection, as the complex forms at DSB ends through the interaction of SHLD2 with >50 nt-long ssDNA^28,31,32,63^. AID-generated DSBs or at least a fraction of them are predicted to contain either 5′ or 3′ overhangs of variable lengths that offer an ideal substrate for SHLD. By protecting AID-DSB ends against resection, we propose that SHLD permits ssDNA processing and end-joining—by NHEJ or alt-NHEJ—to take place within switch regions, leading to productive CSR. The proliferative nature of switching B cells might also account for the need to protect AID-DSB ends, specifically in the S/G2-M phase where there is more extensive DNA end resection^64^. In that regard, while NHEJ dominates in G1, alt-NHEJ acts predominantly in S-G2/M where it is associated with extensive resection and frequent microhomologies at junctions^9,64–66^. Thus, SHLD-mediated DNA end protection might become increasingly essential for productive CSR in NHEJ-deficient cells that rely on alt-NHEJ for the repair of AID-DSBs. Consistent with this, we find that while NHEJ-proficient SHLD1 knockout B cells still perform substantial CSR (approximately 15% of WT cells depending on the switched isotype), XLF or XRCC4 knockout B cells strictly rely on SHLD1 to perform CSR with an almost complete absence of functional switched products (*i.e*. those harboring breakpoint junctions within switched regions) in activated XLF/SHLD1- or XRCC4/SHLD1-deficient B cells. End-joining is however not abolished in these cells as we find that activated NHEJ/SHLD1-deficient B cells contain numerous non-functional switched products containing very long resection and microhomologies at the junctions. Thus, we propose that by limiting the action or the kinetics of the resection machinery(ies), the 53BP1-SHLD axis does not simply promote NHEJ but ensures the fidelity of both NHEJ and alt-NHEJ pathways to permit productive CSR in rapidly dividing activated B cells. This model is consistent with previous work suggesting that 53BP1 acts primarily to ensure the fidelity of DSB repair^24,67^. In this view, the dynamic nature of SHLD complex assembly and disassembly would be an important parameter to allow for multiple DSB repair machineries to accurately process and join DSB ends^56,68,69^. The nature of the alt-NHEJ pathway acting during CSR remains to be fully established^10,11,23^. First, alt-NHEJ associated with relatively short-resection tracks in NHEJ-deficient B cells might differ from alt-NHEJ associated with extensive resection in SHLD/NHEJ-deficient cells. Second, distinct alt-NHEJ sub-pathways might operate depending on the type of NHEJ deficiency present in the cell. For instance, CSR products recovered from Ku-deficient B cells differ from those recovered from XRCC4- or Lig4-deficient cells^70^. In that regard, Rad52 competes with Ku for S-region DSB end binding in wild-type cells and promotes CSR in Ku80 knockdown B cells^71^. Interestingly, Rad52 also mediates IgD CSR by microhomology-mediated alternative end-joining^72^. Whether the SHLD complex is important for Rad52-dependent CSR remains to be fully investigated. Regardless of the nature of alt-NHEJ, our findings predict that the SHLD complex promotes its fidelity for the benefit of productive CSR. SHLD1 being the most recently evolved component of the DSB response apparatus that co-emerged with CSR in vertebrates^29^, it thus could have provided an ultimate evolutionary-selected brick to a complex protein network that led to the emergence of antibody class switching.

## Methods

### Mice

*Shld1*^*+/−*^ *Xlf*^*−/−*^ mice were bred with *Shld1*^*+/−*^ *Xlf*^*−/−*^ mice, and *Shld1*^*−/−*^ *Xlf*^*+/−*^ with *Shld1*^*−/−*^ *Xlf*^*+/−*^ to generate doubly deficient mice. *Shld1*^*−/−*^ *Xrcc4*^*f/f*^ mice were crossed to *Shld1*^*−/−*^ *CD21-cre*^*Tg*^ *Xrcc4*^*f/+*^ to generate *Shld1*^*−/−*^ *CD21-cre*^*Tg*^ *Xrcc4*^*f/f*^ mice. Genotyping of these mutants was performed by PCR of tail DNA. Potential CRISPR/Cas9 off-targets in *Shld1*^*−/−*^ mice were assessed by PCR of B cells genomic DNA and sequencing (see Supplementary Table 7 for primers sequences). The *Xlf*^*−/−*^ ^39^, *Atm*^*−/−*^ ^47^, *53bp1*^*−/−*^ ^73^, *Xrcc4*^*f/f*^ ^44^, and *CD21-Cre*^*Tg*^ ^45^ mice were used in previous studies. Mice were bred under specific-pathogen-free (SPF) conditions and housed at ambient temperature and humidity with 12 h light/12 h dark cycles. In all experiments, 6–17-week-old sex- and age-matched littermates were used. Mice euthanasia was performed by carbon dioxide exposure. All experiments were performed after authorization was granted by the institutional animal care and ethical committee of Institut Pasteur/CETEA n°89 under protocol numbers 180006/14778.

### Lymphocyte development

Lymphocyte development was analyzed in the thymus, bone marrow, and spleen from 6- to 17-week-old sex- and age-matched mice. All single-cell suspensions were treated with Fc-blocking antibody (CD16–32, BD Biosciences 553142, clone 2.4G2, 1:200 dilution) before cell surface staining, which was performed in phosphate-buffered saline (PBS) with 2% fetal bovine serum for 20 min at 4 °C. Bone marrow B lineage cell populations were identified based on the expression of the following markers: pro-B (B220^lo^ CD43^+^ CD19^+^ IgM^−^), pre-B (B220^lo^ CD43^−^ CD19^+^ IgM^−^), immature B cells (B220^lo^ IgM^+^), recirculating B cells (B220^hi^ IgM^+^). T lineage cell populations from the thymus were identified based on the expression of the following markers: double-negative (DN) cells (CD4^−^CD8^−^), DN1 (CD4^−^CD8^−^CD44^+^CD25^−^), DN2 (CD4^−^CD8^−^CD44^+^CD25^+^), DN3 (CD4^−^CD8^−^CD44^−^CD25^+^), DN4 (CD4^−^CD8^−^CD44^−^CD25^−^), double-positive (DP) cells (CD4^+^CD8^+^) and single-positive (SP) cells (CD4^+^CD8^−^ and CD4^−^CD8^+^). Lymphocytes from the spleen were identified based on the expression of the following markers: total B cells (CD19^+^IgM^+^), Marginal Zone B cells (B220^+^CD93^−^CD23^−^CD21^high^), Follicular B cells (B220^+^CD93^−^CD23^+^CD21^+^), and T cells (CD3^+^TCRβ^+^). The following antibodies were used for cell surface staining: anti-B220 (557669, clone RA3–6B2, 1:200 dilution), anti-CD43 (553271, clone S7, 1:150 dilution), CD19 (560375, clone 1D3, 1:200 dilution), anti-IgM (552867, clone R6–60.2, 1:150 dilution), anti-CD93 (558039, clone AA4.1, 1:200 dilution), anti-CD23 (562929, clone B3B4, 1:200 dilution), anti-CD21 (561770, clone 7G6, 1:500 dilution), anti-CD4 (553048, clone RM4–5, 1:200 dilution), anti-CD8a (557668, clone 53–6.7, 1:200 dilution), anti-CD3e (553066, clone 145-2C11, 1:200 dilution), anti-CD44 (560451, clone IM7, 1:200 dilution), anti-CD25 (552880, clone PC61, 1:200 dilution), and anti-TCRβ (47-5961-82, clone H57–597, 1:200 dilution). All antibodies were purchased from BD Biosciences except anti-TCRβ, which was from eBiosciences. Flow cytometry was performed on a FACS Fortessa (BD Bioscience) and data were analyzed using FlowJo v10.4.2 (TreeStar). The gating strategy is provided in Supplementary Fig. 8.

### Generation and culture of *v-Abl* transformed pro-B and CH12 cell lines

*v-Abl* pro-B cell lines were generated as previously described^40^. Briefly, total bone marrow from WT and *Shld1*^*−/−*^ 7-week-old mice was cultured and infected with a retrovirus encoding *v-Abl* kinase to generate immortalized pro-B cell lines^40^. *v-Abl* transformed pro-B cell lines were then transduced with pMSCV-Bcl2-puro retrovirus^40^ to protect them from *v-Abl* kinase inhibitor-induced cell death. *v-Abl* pro-B cells were maintained in RPMI 1640 (Gibco 61870044) supplemented with 10% fetal bovine serum (Sigma F6178), penicillin (100 U/ml)/streptomycin (100 μg/ml) (Gibco) and 50 μM 2-mercaptoethanol (Gibco 11528926).

CH12 cells were cultured in RPMI 1640 supplemented with 12% FBS (Sigma F6178), penicillin (100 U/ml)/streptomycin (100 μg/ml), 50 μM 2-mercaptoethanol, 1x MEM nonessential amino acids (Gibco 12084947), 1 mM sodium pyruvate (Gibco 11530396), and 10 mM HEPES (Gibco 11560496).

### CRISPR/Cas9 editing of *v-Abl* pro-B and CH12 cell lines

*v-Abl* pro-B and CH12 knockout cell clones were generated as previously described^32,40,41^. Briefly, cells were nucleofected with two sgRNA-encoding plasmids and the pCas9-GFP plasmid (see Supplementary Table 7 for sgRNAs sequences) using an Amaxa Nucleofector and Nucleofector Kit V solution (Lonza). Electroporated cells were left to recover for 36–48 h and single cells expressing GFP were sorted in 96-well plates. Clones were then screened by PCR (see Supplementary Table 7 for primers sequences), and purified PCR products were Sanger sequenced to identify CRISPR/Cas9-induced deletions and insertions at the cleavage site (see Supplementary Table 8). Knockout clones were then validated by Western blot (Supplementary Fig. 9).

### V(D)J recombination assay

V(D)J recombination assays were performed as previously described^38,40^. Briefly, *v-Abl* pro-B cells were transduced with pMX-INV retroviral vector and cells that had integrated the recombination substrate were enriched based on hCD4 expression using hCD4 Microbeads (Miltenyi 130-045-101). Purified *v-Abl* infected pro-B cells (10^6^ per ml) were treated with 3 μM of the *v-Abl* kinase inhibitor STI571 (Novartis) for 72 h and assayed for rearrangement levels by FACS analysis of GFP/hCD4 expression (hCD4-PE antibody, Miltenyi 130-113-254, clone M-T466, 1:100). V(D)J recombination efficiency was scored as the percentage of GFP-positive cells among hCD4-positive cells. FACS data was acquired by FACS Diva v8.0.1 (BD Biosciences) and analyzed and visualized by Flowjo v10.4.2 (TreeStar). The gating strategy is provided is Supplementary Fig. 8.

### PCR analysis of endogenous *Igk* rearrangements

Endogenous Vk_6-23_/Jk_1_ coding joints were amplified as previously described^38,74^. 500 ng of genomic DNA was amplified using pkJa2 and pk6c primers. The following parameters were used: 1× (95 °C 5 min); 17× (94 °C 30 s, 60 °C 30 s, and 72 °C 30 s); 1× (72 °C 5 min). Serial fourfold dilutions of this reaction were amplified using pkJa2 and pk6d primers and the following cycles: 1× (95 °C 5 min); 25× (94 °C 30 s, 60 °C 30 s, and 72 °C 30 s); 1× (72 °C 5 min). *Il2* gene was amplified using IMR42 and IMR43 primers and was used as a loading control. The PCR gel images were acquired and analyzed using Image Lab v6.0 (Biorad). See Supplementary Table 7 for primer sequences.

### PCR analysis of *TCRβ* rearrangements

100, 50, and 10 ng of gDNA from thymocytes was amplified using either TCRB-D1US and TCRB-J1D-A (Dβ1–Jβ1), or TCRB-D2US and TCRB-J2D-A (Dβ2-Jβ2). PCR reactions (20 μl) contained genomic DNA template, 0.5 μM of each primer, and 1X Platinum SuperFi II PCR Master Mix (Invitrogen 16445389). The following parameters were used: 1× (98 °C 3 min); 35× (98 °C 45 s, 60 °C 90 s, and 72 °C 150 s); 1× (72 °C 10 min). *Il2* gene was amplified using IMR42 and IMR43 primers and was used as a loading control. The PCR gel images were acquired and analyzed using Image Lab v6.0 (Biorad). See Supplementary Table 7 for primer sequences.

### Quantitative PCR analysis of *TCRα/δ* rearrangements

100 ng of gDNA from thymocytes was amplified with a combination of gene-specific primers (see Supplementary Table 7 for primers sequences). PCR reactions (50 μl) contained genomic DNA template, 0.4 μM of each primer and 1X Power Sybr Green Master Mix (Applied Biosystems 4367659). Quantitative PCR was performed in triplicates and data were collected on a QuantStudio 3 Real-Time PCR System and analyzed using the QuantStudio Design & Analysis Software v2.6.0. For each assay, aliquots of DNA were analyzed for a control, non-rearranging DNA 3′ of Jδ2. The cycle threshold numbers for each primer combinations (*C*_t_^exp^) and for the control amplification (*C*_t_^ctr^) were used to calculate the absolute amount of PCR signal. The relative ratios of each rearrangement were averaged and plotted together with the standard error of the mean. See Supplementary Table 7 for primer sequences.

### Ex vivo CSR assay

Splenic B cells were purified from 6–17-week-old sex- and aged-matched mice using magnetic CD19 beads according to the manufacturer’s instructions (Miltenyi Biotec 130-121-301). In total, 2.4 × 10^6^ cells were cultured in 4 ml of complete medium consisting of RPMI 1640 supplemented with 12% FBS, penicillin (100 U/ml)/streptomycin (100 μg/ml), 50 μM 2-mercaptoethanol, and incubated at 37 °C in a humidified atmosphere containing 5% CO_2_. To induce specific isotype switching, B cells were stimulated with either LPS (25 μg/ml, Sigma-Aldrich), IL-4 (10 ng/ml, Miltenyi) and anti-IgD dextran (3 ng/ml, Fina Biosolutions) or anti-CD40 antibody (1 μg/ml, Miltenyi) and IL-4 for IgG1, and LPS for IgG2b and IgG3. Cells incubated with either anti-IgD dextran or anti-CD40 antibody were used as a negative control. After 4 to 5 days, cells were assayed for class switching by flow cytometry using CD19-V450 (BD Biosciences 560375, clone 1D3, 1:200 dilution), B220-A488 (BD Biosciences 557669, clone RA3–6B2, 1:200 dilution), B220-APC (BD Biosciences 553092, clone RA3–6B2, 1:200 dilution), IgG1-APC (BD Biosciences 550874, clone X56, 1:500 dilution), IgG2b-PE (Biolegend 406708, clone RMG2b-1, 1:500 dilution), IgG3-FITC (BD Biosciences 553403, clone R40-82, 1:500 dilution), IgM-PE-Cy7 (BD Biosciences 552867, clone R6–60.2, 1:200 dilution), Thy1.1-BV421 (BD Bioscience 740044, clone HIS51, 1:300 dilution) antibodies and a Fortessa analyzer (BD Biosciences). Data were analyzed by FlowJo v10.4.2 software. The gating strategy is provided in Supplementary Fig. 8. Viable cells were counted using a Casy cell counter (Roche). Where indicated, cells were incubated with DMSO or 2.5μM ATM inhibitor (Calbiochem 118502). For analysis of SHLD1 domains, cells were infected with retroviral-based vectors expressing wildtype and mutant forms of SHLD1 after 1 to 2 days of stimulation. For proliferation analysis by in vitro cell labeling, purified B cells were stained at 5 × 10^6^ cells/mL in PBS with 5μM Cell Trace^TM^ violet (CTV) for 8 min at room temperature (Thermo Scientific 10220455), following the standard protocol with two additional washes with the medium. CTV-stained B cells were incubated for 30 min at 37 °C and then stimulated with LPS, IL-4, and anti-IgD dextran as described above.

### CSR assay in CH12 cell lines

CH12 cells were plated at 50,000 cells per ml in complete RPMI supplemented with anti-CD40 antibody (1 μg/ml, Miltenyi), IL-4 (20 ng/ml, Miltenyi), and TGF-β (1 ng/ml, R&D Biotech) to induce IgM to IgA switching. After 3 days, cells were assayed for class switching by flow cytometry using IgA-PE (eBiosciences 12-4204-82, clone mA-6E1, 1:200) and IgM-PE-Cy7 (BD Biosciences 552867, clone R6–60.2, 1:200 dilution) antibodies, and a Fortessa analyser (BD Biosciences). Data were analyzed by FlowJo v10.4.2 software. The gating strategy is provided in Supplementary Fig. 8.

### Immunoprecipitation

Nuclear extracts were prepared by resuspending 40 million fresh cells in ice-cold 10 ml buffer A (10 mM HEPES pH 7.9, 10 mM KCl, 1.5 mM MgCl_2_, 0.1% NP-40, protease inhibitors cocktail, phosSTOP) and rotating for 10 min at 4 °C. Nuclei were centrifuged at 800×*g* for 10 min at 4 °C and resuspended in 1 ml IP buffer C150 (20 mM HEPES pH 7.9, 150 mM NaCl, 1.5 mM MgCl_2_, 0.2 mM EDTA, 0.25% NP-40, 10% glycerol, protease inhibitors cocktail, phosSTOP). Lysates were briefly sonicated followed by Benzonase (Sigma E8263) digestion for 30 min at 4 °C. Finally, lysates were cleared through centrifugation at 13,000×*g* for 20 min before incubation with 50 μl of anti-Flag M2 magnetic beads (Sigma M8823) overnight at 4 °C. Beads were washed five times in an IP buffer. Washed beads were directly resuspended in Sample Buffer Laemmli 2x (Sigma S3401), and boiled at 95 °C for 10 min.

### DNA–FISH on metaphase spreads

DNA–FISH was performed as previously described^32,38^. Slides were treated with RNase A for 40 min, dehydrated in 70, 90, and 100% ethanol for 3 min each, denatured in 70% formamide/2× SSC for 3 min at 77 °C, dehydrated again in cold ethanol series, and hybridized with probes overnight at 37 °C in a humid chamber. The next day, slides were washed three times in 50% formamide/0.5× SSC for 5 min each at 37 °C and twice in 0.5× SSC for 10 min and 20 min at 37 °C. Finally, slides were mounted in ProLong Gold antifade reagent with DAPI (Invitrogen P36931) to counterstain total DNA. Metaphases were imaged using a ZEISS AxioImager.Z2 microscope and the Metafer automated capture system (MetaSystems) and counted manually. Probes used in this study: *Igh C* BAC probes (RP24–134G24), *Igh V* BAC probe (RP24–386J17), and XCyting Mouse Chromosome 12 paint (MetaSystems). Analysis of the percentage of aberrant metaphases was performed using Microsoft Excel v16.16.2.

### Long-range PCR and sequencing

Long‐range PCR of the Ighμ‐Ighγ1 amplicon was accomplished using Platinum SuperFi II polymerase (Invitrogen 16445389). A total of 100 ng of gDNA was amplified using EμF and Cγ1R2 primers and the following cycles: 1× (98 °C 30 s); 30× (98 °C 10 s, 57 °C 10 s, and 72 °C 8 min); 1× (72 °C 5 min). See Supplementary Table 7 for primer sequences.

Prior to library preparation, all amplicons were purified using Ampure PB beads at 1.8X and controlled using a Fragment Analyzer with a High Sensitivity gDNA kit. Amplicon Sequencing library was prepared according to the Pacific Biosciences recommendations (protocol version: Part Number 101-791-700 version 06 (January 2021)). We sequenced PCR products obtained from three replicates of wildtype and mutant splenic B cells using long-read PacBio Sequel I technology. For each of them, three replicates were included accounting for a total of 12 samples that were multiplexed in one sequencing run. The resulting sequences were demultiplexed and consensus circular reads (CCS) were constructed using the SMRTLINK software from PacBio (v9) (smrtlink: https://www.pacb.com/support/software-downloads/). Then, CCS reads were converted into FastQ files and mapped onto the Mus musculus genome (reference GRCm38 v92) using minimap2^75^ including PacBio and splicing options. Subsequent bioinformatics analysis of the aligned reads was performed with the Sequana library (BAM file analysis, visualization)^76^. We filtered out reads that do not align on both 5’ and 3’ sections of the region of interest (chromosome 12, 113,326,440–113,427,620).

### RT-PCR

*Igh γ1* and *γ3* germline transcripts (GLT) and *Aid* mRNA expression were assessed as previously described^32^ by semi-quantitative RT-PCR using 2.5-fold serial dilutions of cDNA from primary cells stimulated for 4 to 5 days with LPS and IL-4, or LPS only. *Hprt* was used as a control for transcript expression. Primer sequences are listed in Supplementary Table 7.

### Western blotting

Primary cells were lysed using Cell lysis buffer (10 mM HEPES pH = 7.5, 10 mM KCl, 0,2 mM EDTA, 1 mM DTT, 0,5% NP-40, protease inhibitors cocktail, 300 mM sucrose) and 20 min incubation on ice. Nuclei were centrifuged at 650×*g* for 10 min at 4 °C and resuspended in Nuclear extraction buffer (20 mM HEPES pH 7.5, 420 mM NaCl, 1 mM EDTA, 1 mM DTT, 0.5% NP-40) for 30 min on ice. Lysates were briefly sonicated and then cleared through centrifugation at 13,000×*g* for 15 min. CH12 and pro-B cells were lysed using RIPA cell lysis reagent (Thermo Fisher Scientific 89900) and protease inhibitors cocktail (Roche 11873580001). Equal amounts of proteins were subjected to SDS-PAGE on 4–12% Bis-Tris gel (Invitrogen). Proteins were transferred onto a nitrocellulose membrane (Invitrogen) using the iBlot apparatus (P3 program, 7 min transfer, Invitrogen). Membranes were incubated in 5% non-fat dried milk in TBS containing 0.1% Tween-20 buffer for at least 1 h at room temperature and subsequently incubated overnight at 4 °C with primary antibody (SHLD1: Thermo Fisher Scientific PA5-559280, 1:200; XLF: Bethyl Laboratories A300-729A, 1:1000; 53BP1: Novus Biologicals NB100-304, 1:1000; MAD2L2: Protein Tech 12683-1-AP, 1:500; FLAG: Protein Tech 20543-1-AP, 1:2000). γ-Tubulin (Sigma-Aldrich T6557, clone GTU-88, 1:10000) or Lamin B (Abcam 16048, 1:2000) was used as a loading control. HRP-conjugated anti-mouse secondary antibody (Cell Signaling Technology 7076, 1:10000) or HRP-conjugated anti-rabbit secondary antibody (Cell Signaling Technology 7074, 1:5000) were used. Immune complexes were detected with Clarity Western ECL Substrate (Biorad 170-5060) or WesternBright Sirius substrate (Advansta K12043-D10). Blots were developed and analyzed using Image Lab v6.0 (Biorad). For XRCC4 blots, the membranes were incubated in Intercept (TBS) Blocking Buffer (LI-COR) for 1 h at room temperature and incubated successively with XRCC4 (Santa Cruz sc-8285, clone C20, 1:1300) and γ-Tubulin primary antibodies. IRDye-conjugated anti-goat (LI-COR IRDye^®^ 800CW Donkey anti-Goat IgG, 926-32214, 1:10,000) and anti-mouse (LI-COR IRDye^®^ 680RD Goat anti-Mouse IgG, 926-68070, 1:20,000) secondary antibodies were used. Immune complexes were detected using LI-COR Odyssey Imaging System.

### ELISAs

High-binding 96-well ELISA plates (Costar, Corning) were coated overnight with goat anti-mouse IgGs, anti-mouse IgMs (Jackson ImmunoResearch), or anti-mouse IgG1 antibodies (Southern Biotech) (250 ng/well in PBS). Plates were washed with 0.05% Tween-20-PBS (PBST), blocked for 2 h with 2% BSA, and 1 mM EDTA-PBST (Blocking buffer). After PBST-washings, plates were incubated for 2 h with 1:100 PBS-diluted plasma and seven consecutive 1:3 dilutions in duplicate. Purified mouse IgG, IgG1 (Sigma-Aldrich), and IgM (Merck Millipore) antibodies starting at 12 µg/ml and seven consecutive 1:3 dilutions in PBS were used as standards. After PBST-washings, plates were incubated for 1 h with HRP-conjugated goat anti-mouse IgGs, anti-mouse IgM (Jackson ImmunoResearch), or rat anti-mouse IgG1 antibodies (Southern Biotech) in blocking buffer, washed, and revealed by adding HRP chromogenic substrate (ABTS solution, Euromedex). Experiments were performed at room temperature using HydroSpeed™ microplate washer and Sunrise™ microplate absorbance reader (Magellan v7.2, Tecan Männedorf), with optical density measurements made at 405 nm (OD405 nm).

### CSR-HTGTS

HTGTS libraries with a 5′Sμ bait were generated from primary splenic B cells stimulated with LPS, IL-4, and anti-IgD dextran for 96 h. A total of 25 μg gDNA from the stimulated splenic B cells was sonicated (25 s ON and 60 s OFF, two cycles with low-energy input) on a Diagenode Bioruptor sonicator. The templates were amplified with a biotinylated 5′Sμ primer after sonication. The LAM-PCR products were incubated with streptavidin C1 beads (#65001, Thermo Fisher Scientific) for 4 h at room temperature. The enriched biotin-labeled LAM-PCR products were ligated with an adapter with the following program (25 °C 1 h, 22 °C 3 h, 16 °C overnight). The adapter-ligated products were amplified by nested PCR with barcode primers and tag PCR with P5-I5 and P7-I7 primers. The PCR products from 500 bp to 1000 bp were purified by separation on 1% TAE gel. HTGTS libraries were sequenced by paired-end 150 bp sequencing on a Next-SeqTM550 (Illumina). More details of the method have been described^14,77^.

Libraries were processed via the published pipeline^78^. The libraries from *Shld1*^*−/−*^, *Xlf*^*−/−*^, *Xlf*^*−/−*^*Shld1*^*−/−*^ and their WT C57BL/6 control mice were mapped against the mm9 genome as described previously^14,77^. Data were analyzed and plotted after removing duplicates^14,77^. Each experiment was repeated three times for statistical analyses. The junction numbers within the Sμ and Sγ1, as well as the percentage analysis of these S-regions with respect to total junctions within the C_H_-containing portion of the IgH were plotted in Fig. 5 and Supplementary Fig. 7. The numbers and percentages were indicated in the corresponding panels. The MH analysis were described in a previous study^13^.

### Statistics and reproducibility

Statistical analyses were performed with GraphPad Prism v7.04 and BiostaTGV (https://biostatgv.sentiweb.fr/) and using the tests as indicated in the respective figure legends. Significance; ns, not significant (*p* ≥ 0.05), *p < 0.05, **p < 0.01, ***p < 0.001, ****p < 0.0001. Unless indicated otherwise, all immunoblots are representative of at least two independent experiments, with uncropped blots shown in the Source data file.

### Reporting Summary

Further information on research design is available in the Nature Research Reporting Summary linked to this article.

## Supplementary information

Supplementary Information

Reporting Summary

## Data Availability

The CSR-HTGTS data have been deposited in NCBI’s Gene Expression Omnibus under the accession number GSE202567. The Long-range PCR sequencing (Pacbio) data have been deposited in NCBI’s Sequence Read Archive under the accession number PRJNA831666. All other data can be found in the Supplementary Data of this paper or in the Source Data. This includes all uncropped blots, gels, and data shown in graphs throughout the manuscript, including the Supplementary Figures. All data are available from the authors upon reasonable request. Source data are provided with this paper.

## Source data

Source Data

## Code availability

All of the code used in the paper was from published studies and is cited in the manuscript.
