# Supplementary material for: SHLD1 is dispensable for 53BP1-dependent V(D)J recombination but critical for productive class switch recombination
Source: Nat Commun. 2022 Jun 28;13:3707. doi: 10.1038/s41467-022-31287-3 (PMC9240092; doi:10.1038/s41467-022-31287-3)
Supplement: Supplementary file 1 — Supplementary Information [file 41467_2022_31287_MOESM1_ESM.pdf]

Supplementary information

SHLD1 is dispensable for 53BP1-dependent V(D)J recombination but critical for productive class switch recombination

Estelle Vincendeau, Wenming Wei, Xuefei Zhang, Cyril Planchais, Wei Yu, Hélène Lenden-Hasse, Thomas Cokelaer, Juliana Pipoli da Fonseca, Hugo Mouquet, David J. Adams, Frederick W. Alt, Stephen P. Jackson, Gabriel Balmus, Chloé Lescale and Ludovic Deriano.

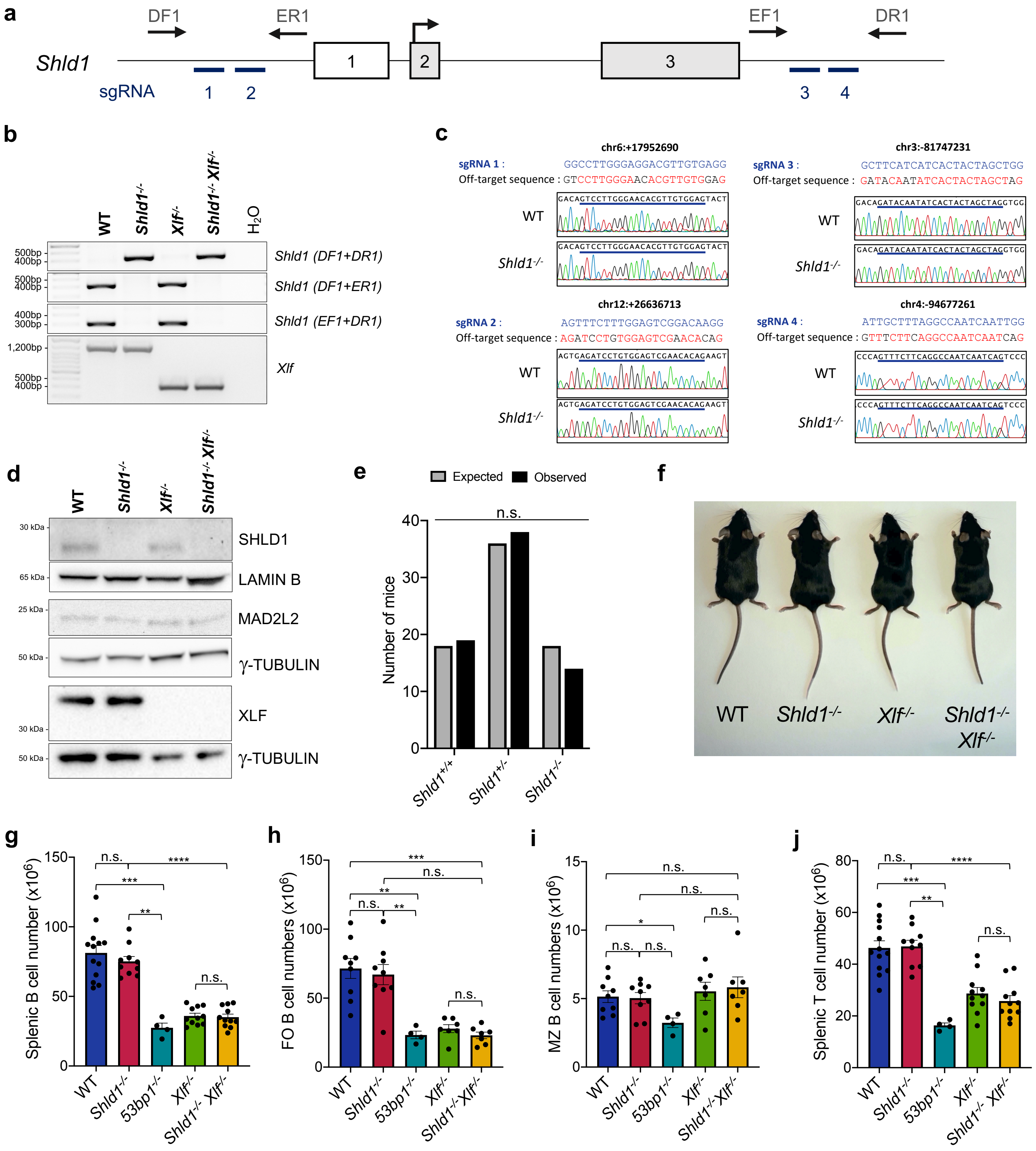

Supplementary Figure 1 (related to Figure 1). *Shld1$^{-/-}$* mice are viable and show no overt developmental phenotype. a CRISPR/Cas9 *Shld1* knockout strategy. Exons are shown in boxes, and sgRNAs positions are indicated. Arrows show primers used for genotyping. **b** PCR analysis showing the deletion of the complete *Shld1* coding sequence (64572 bp) and *Xlf*. *Shld1* PCR products sizes : DF1 + DR1 : 426 bp; DF1 + ER1 : 414 bp; EF1 + DR1 : 301 bp. *Xlf* PCR products sizes: WT: 1232bp; KO: 445bp. **c** Sequencing analysis of sgRNAs off-target sites in WT and *Shld1$^{-/-}$* mice. **d** Western blot analysis of SHLD1, MAD2L2 and XLF expression in splenocytes from WT, *Shld1$^{-/-}$*, *Xlf$^{-/-}$* and *Shld1$^{-/-}$ Xlf$^{-/-}$* mice. LAMIN B and γ-TUBULIN are used as loading controls. **e** Number of live-born mice obtained from crosses between *Shld1$^{+/-}$* mice. Expected versus observed numbers were used to calculate one-sided Chi-square. **f** Picture of 10-12 weeks-old WT, *Shld1$^{-/-}$*, *Xlf$^{-/-}$* and *Shld1$^{-/-}$Xlf$^{-/-}$* mice. **g** Number of splenic CD19^{+}IgM^{+} total B cells. Bars represent mean ± s.e.m., n = 13 (WT), n = 10 (*Shld1$^{-/-}$*), n = 4 (*53bp1$^{-/-}$*), n = 11 (*Xlf$^{-/-}$*; *Shld1$^{-/-}$ Xlf$^{-/-}$*), two-sided Wilcoxon–Mann–Whitney test (***p = 0.0008; **p = 0.002; ****p < 0.0001). Each dot represents a mouse. **h** Number of splenic follicular (FO) B cells. Bars represent mean ± s.e.m., n = 9 (WT; *Shld1$^{-/-}$*), n = 4 (*53bp1$^{-/-}$*), n = 7 (*Xlf$^{-/-}$*; *Shld1$^{-/-}$ Xlf$^{-/-}$*), two-sided Wilcoxon–Mann–Whitney test (**p = 0.0028 (WT vs *53bp1$^{-/-}$*); ***p = 0.0002 (WT vs *Shld1$^{-/-}$ Xlf$^{-/-}$*); **p = 0.0056 (*Shld1$^{-/-}$* vs *53bp1$^{-/-}$*)). Each dot represents a mouse. **i** Number of splenic marginal zone (MZ) B cells. Bars represent mean ± s.e.m., n = 9 (WT; *Shld1$^{-/-}$*), n = 4 (*53bp1$^{-/-}$*), n = 7 (*Xlf$^{-/-}$*; *Shld1$^{-/-}$ Xlf$^{-/-}$*), two-sided Wilcoxon–Mann–Whitney test (*p = 0.0196). Each dot represents a mouse. **j** Number of splenic CD3^{+}TCRβ^{+} T cells. Bars represent mean ± s.e.m., n = 13 (WT), n = 10 (*Shld1$^{-/-}$*), n = 4 (*53bp1$^{-/-}$*), n = 11 (*Xlf$^{-/-}$*; *Shld1$^{-/-}$ Xlf$^{-/-}$*), two-sided Wilcoxon–Mann–Whitney test (***p = 0.0008 (WT vs *53bp1$^{-/-}$*); **p = 0.002 (*Shld1$^{-/-}$* vs *53bp1$^{-/-}$*); ****p < 0.0001)). Each dot represents a mouse. n.s. : non-significant (p ≥ 0.05), *p<0.05, **p<0.01, ***p<0.001, ****p<0.0001. Source data are provided as a Source Data file.

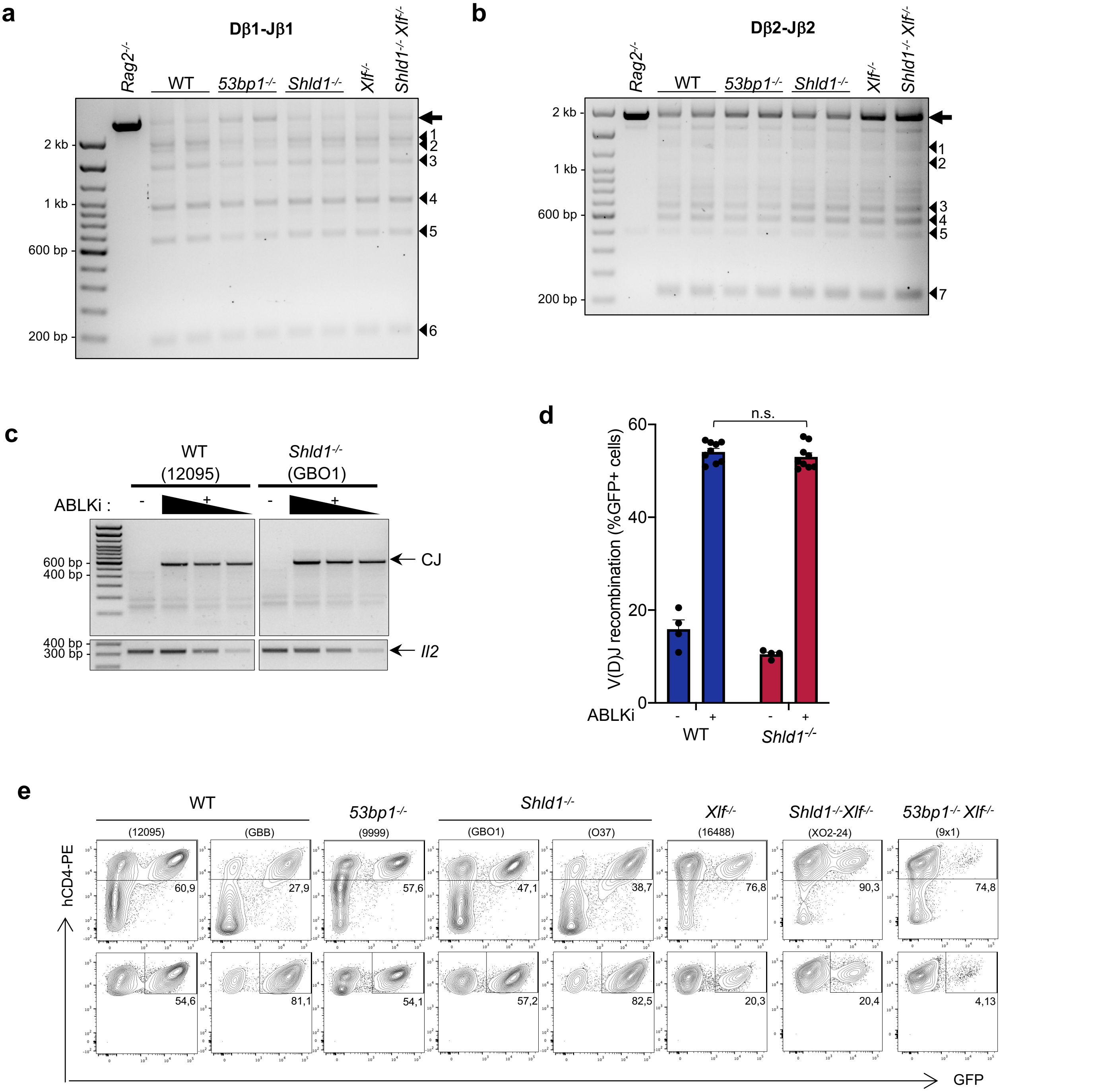

Supplementary Figure 2 (related to Figure 2). 53BP1 displays SHLD1-independent functions during V(D)J recombination. **a** PCR analysis of Dβ1 to Jβ1 rearrangements in *Rag2*$^{-/-}$, WT, *Shld1*$^{-/-}$, *53bp1*$^{-/-}$, *Xlf*$^{-/-}$ and *Shld1*$^{-/-}$*Xlf*$^{-/-}$ thymocytes. **b** PCR analysis of Dβ2 to Jβ2 rearrangements. **c** Semi-quantitative nested PCR analysis of $Igk_{6\text{-}23}$ –J_1 coding joint (CJ) in *v-Abl* pro-B cell lines treated for 72h with ABLKi. *Il2* gene PCR was used as a loading control. **d** Bar plot showing V(D)J recombination efficiency of pMX-INV recombination substrate in *v-Abl* pro-B cell lines. *v-Abl* pro-B cell lines of each genotype were treated with ABLKi for 72h and GFP$^+$ cells were analyzed by flow cytometry. Bars represent mean ± s.e.m, two-sided Wilcoxon–Mann –Whitney test, n=4 independent experiments with duplicates of ABLKi treated cells, (WT (clone #12095) and *Shld1*$^{-/-}$ (clone #GBO1)). **e** Representative flow cytometry analysis of V(D)J recombination in *v-Abl* pro-B cells clones. n.s. : non-significant (p ≥ 0.05). Source data are provided as a Source Data file.

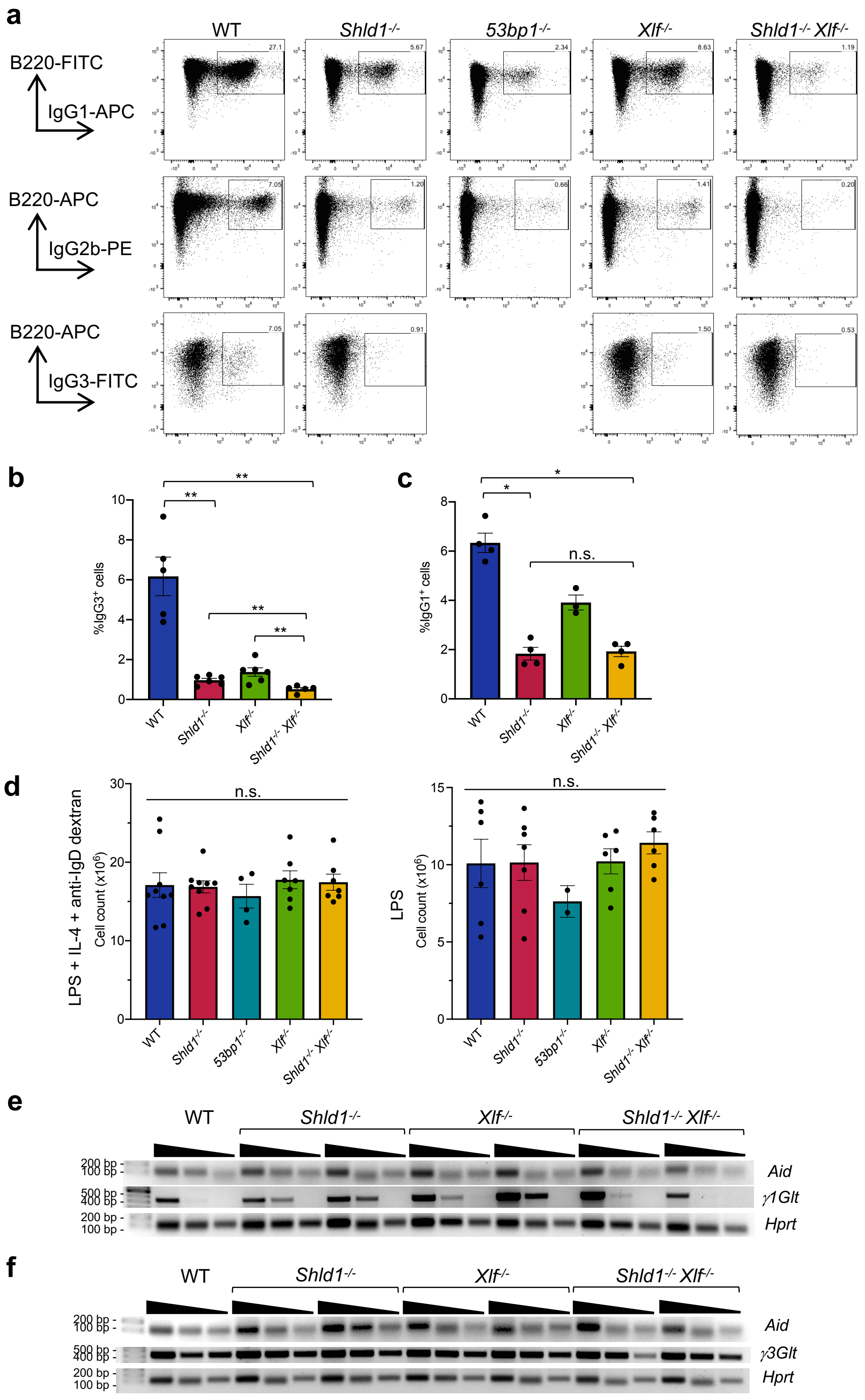

Supplementary Figure 3 (related to Figure 3). No defect in cell proliferation, *Aid* expression or *Igh* germline transcripts levels in stimulated *Shld1/Xlf*-deficient B cells a Surface expression of IgG1, IgG2b and IgG3 isotypes on *in vitro*–stimulated WT, *Shld1*$^{-/-}$, *53bp1*$^{-/-}$, *Xlf*$^{-/-}$ and *Shld1*$^{-/-}$*Xlf*$^{-/-}$ B cells. Cells were harvested after 4 days of *in vitro* stimulation with anti-IgD dextran, LPS and IL-4 (IgG1) or LPS (IgG2b and IgG3), and flow cytometric analysis was used to determine surface IgG1, IgG2b and IgG3 expression. Numbers on the dot plots show the percentage of switched cells as a proportion of B220+ B cells. **b** Percentages of WT, *Shld1*$^{-/-}$, *Xlf*$^{-/-}$ *and Shld1*$^{-/-}$ *Xlf*$^{-/-}$ B cells expressing IgG3 isotype after 4 days *in vitro*–stimulation with LPS. Bars represent mean ± s.e.m., n = 5 except for *Shld1*$^{-/-}$ and *Shld1*$^{-/-}$*Xlf*$^{-/-}$ (n = 6), two-sided Wilcoxon–Mann–Whitney test (**p = 0.0043 (WT vs *Shld1*$^{-/-}$; *Xlf*$^{-/-}$ vs *Shld1*$^{-/-}$*Xlf*$^{-/-}$); **p = 0.0079 (WT vs *Shld1*$^{-/-}$*Xlf*$^{-/-}$); **p = 0.0087 (Shld1$^{-/-}$ vs *Shld1*$^{-/-}$*Xlf*$^{-/-}$)). **c** Percentages of WT, *Shld1*$^{-/-}$, *Xlf*$^{-/-}$ *and Shld1*$^{-/-}$*Xlf*$^{-/-}$ B cells expressing IgG1 isotype after 4 days *in vitro*–stimulation with anti-CD40 antibody and IL-4. Bars represent mean ± s.e.m., n = 4 except for *Xlf*$^{-/-}$ (n = 3), two-sided Wilcoxon–Mann–Whitney test (*p = 0.0286). **d** Cell counts of *in vitro*–stimulated WT, *Shld1*$^{-/-}$, *53bp1*$^{-/-}$, *Xlf*$^{-/-}$ and *Shld1*$^{-/-}$*Xlf*$^{-/-}$ B cells. Cells were plated at 600,000 cells/ml and counted after 4 days stimulation with either anti-IgD dextran, LPS and IL-4 (n = 9 (WT, *Shld1*$^{-/-}$), n = 4 (*53bp1*$^{-/-}$), n = 7 (*Xlf*$^{-/-}$, *Shld1*$^{-/-}$ *Xlf*$^{-/-}$)) or LPS (n = 6 (WT, *Xlf*$^{-/-}$, *Shld1*$^{-/-}$ *Xlf*$^{-/-}$), n = 2 (*53bp1*$^{-/-}$), n = 7 (*Shld1*$^{-/-}$)). Bars represent means ± s.e.m., two-sided Wilcoxon–Mann–Whitney test. **e** *Aid* and *Igh γ1* germ-line transcripts (*γ1Glt*) mRNA were quantified by semi-quantitative RT–PCR using 2.5-fold serial dilutions of cDNA made from B cells after 5 days *in vitro* stimulation with LPS and IL-4. *Hprt* was used as a control for transcript expression. **f** *Aid* and *γ3Glt* mRNA were quantified by semi-quantitative RT–PCR using 2.5-fold serial dilutions of cDNA made from B cells after 5 days *in vitro* stimulation with LPS. *Hprt* was used as a control for transcript expression. n.s. : non-significant (p ≥ 0.05), *p<0.05; **p<0.01. Source data are provided as a Source Data file.

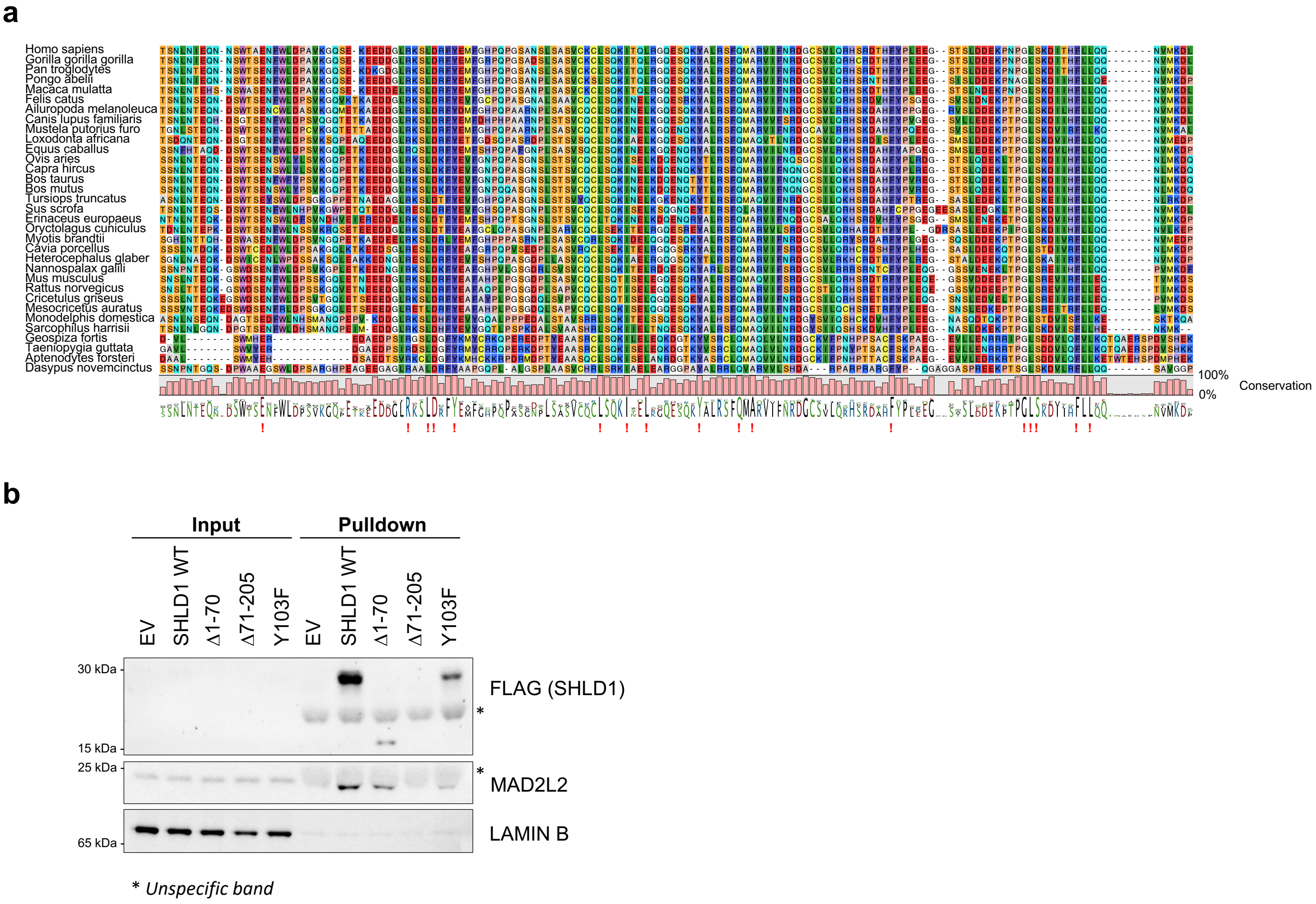

Supplementary Figure 4 (related to Figure 3). Distinct SHLD1 residues mediate its functions during CSR. a Multiple sequence alignment of SHLD1 orthologs corresponding to the position 61 to 205 of human SHLD1. Invariably conserved residues are marked with a red exclamation mark. CLC Main Workbench 20 was used to visualize the alignment result. **b** Flag–SHLD1 WT, Δ1-70, Δ71-205 or Y103F immunocomplexes were isolated from nuclear extracts prepared from untreated complemented *Shld1*$^{-/-}$ pro-B cell cultures. Western blots were probed for the indicated targets. Data are representative of three independent experiments. Source data are provided as a Source Data file.

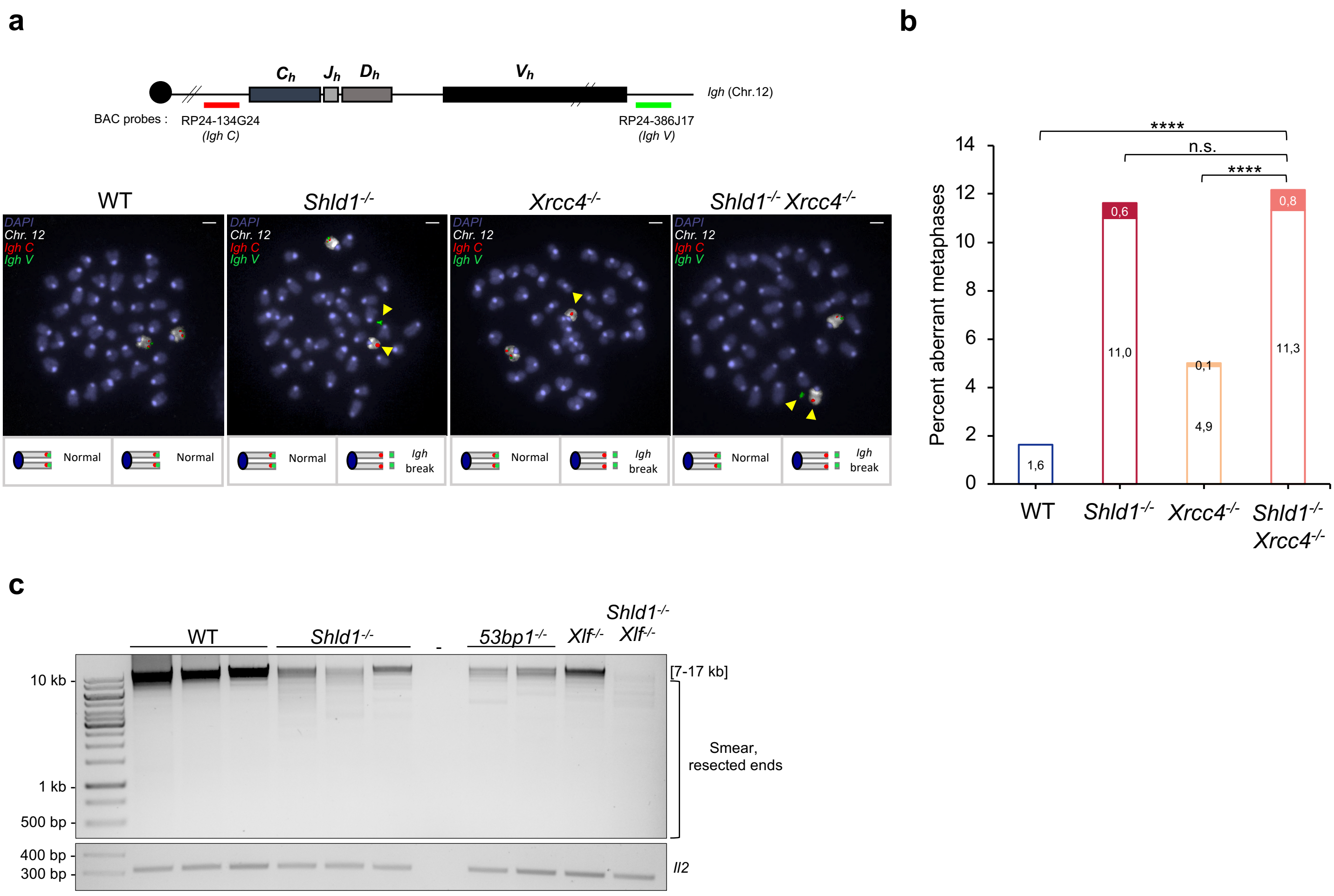

Supplementary Figure 5 (related to Figure 3). SHLD1/53BP1 promote functional CSR in NHEJ-proficient and NHEJ-deficient cells by limiting DNA end resection. a Representative images of *Igh* breaks in aberrant metaphases, as quantified in **b**. Scale bars, 3 µm. **b** Quantification of *Igh* breaks (clear bar) and translocations (filled bar) in metaphases of WT, *Shld1*$^{-/-}$, *Xrcc4*$^{-/-}$ *and Shld1*$^{-/-}$*Xrcc4*$^{-/-}$ cells. Bars represent mean. n = 800 (WT, *Shld1*$^{-/-}$, *Xrcc4*$^{-/-}$), n = 600 (*Shld1*$^{-/-}$ *Xrcc4*$^{-/-}$), two-sided Fisher's exact test (****p<0.0001). See also Supplementary Table 5. **c** Long-range PCR analysis of Sµ to Sγ1 rearrangements in WT, *Shld1*$^{-/-}$, *53bp1*$^{-/-}$, *Xlf*$^{-/-}$ *and Shld1*$^{-/-}$*Xlf*$^{-/-}$ stimulated B cells. *Il2* gene PCR was used as a loading control. n.s. : non-significant (p ≥ 0.05), ***p<0.001. Source data are provided as a Source Data file.

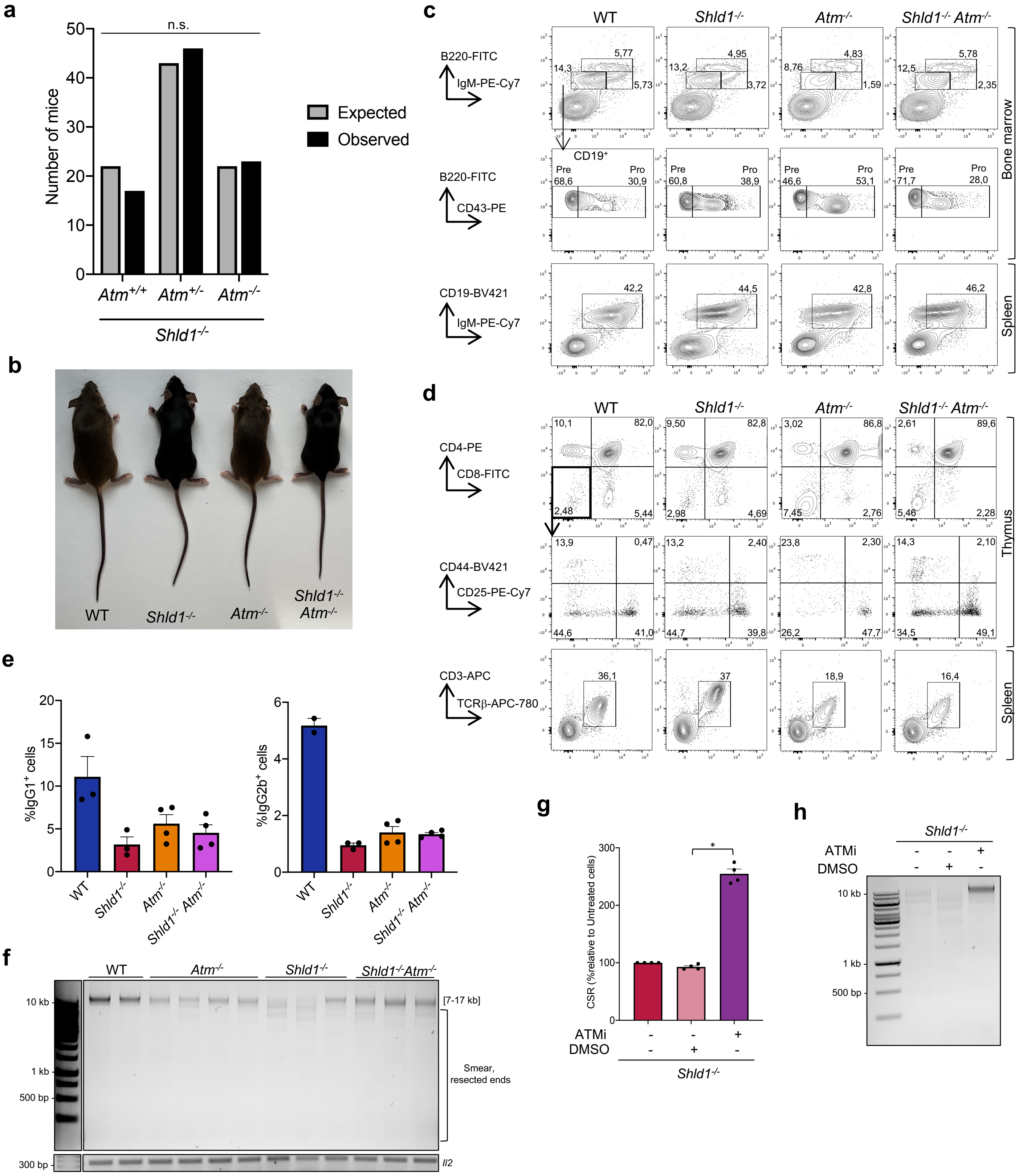

Supplementary Figure 6 (related to Figure 4). ATM might control some of the pro-resection activities in SHLD1-deficient B cells. a Number of live-born mice obtained from crosses between *Shld1*$^{-/-}$ *Atm*$^{+/-}$ mice. Expected versus observed numbers were used to calculate one-sided Chi-square. **b** Picture of 5-6 weeks-old aged-matched WT, *Shld1*$^{-/-}$, *Atm*$^{-/-}$ and *Shld1*$^{-/-}$ *Atm*$^{-/-}$ mice. **c** Representative FACS analysis of bone marrow using B cell markers. Numbers on plots represent percentages of total cells. **d** Representative FACS analysis of thymus using T-cell markers. **e** Percentages of WT, *Shld1*$^{-/-}$, *Atm*$^{-/-}$ and *Shld1*$^{-/-}$*Atm*$^{-/-}$ B cells expressing either IgG1 or IgG2b isotypes after *in vitro*–stimulation. Cells were harvested after 4 days of *in vitro* stimulation with LPS and IL-4 (IgG1) or LPS (IgG2b), and flow cytometric analysis was used to determine surface IgG1 and IgG2b expression. Numbers on the dot plots show the percentage of switched cells as a proportion of CD19^{+} B cells. Bars represent mean ± s.e.m.. IgG1^{+} cells : n = 3 (WT; *Shld1*$^{-/-}$) and n = 4 (*Atm*$^{-/-}$; *Shld1*$^{-/-}$ *Atm*$^{-/-}$); IgG2b^{+} cells : n = 2 (WT), n = 3 (*Shld1*$^{-/-}$) and n = 4 (*Atm*$^{-/-}$; *Shld1*$^{-/-}$ *Atm*$^{-/-}$). **f** Long-range PCR analysis of Sμ to Sγ1 rearrangements in WT, *Atm*$^{-/-}$, *Shld1*$^{-/-}$ and *Shld1*$^{-/-}$*Atm*$^{-/-}$ stimulated B cells. *Il2* gene PCR was used as a loading control. **g** Normalized percentage of IgG1^{+} *Shld1*$^{-/-}$ B cells after *in vitro*–stimulation in the presence or absence of ATMi. Cells were harvested after 4 days of *in vitro* stimulation with LPS, IL-4, anti-IgD dextran and DMSO or ATMi (2.5 μM). Bars represent mean ± s.e.m., n = 4 independent samples, two-sided Wilcoxon–Mann –Whitney test (*p = 0.0286). **h** Long-range PCR analysis of Sμ to Sγ1 rearrangements in *Shld1*$^{-/-}$ stimulated B cells in the presence or absence of ATMi (2.5 μM). *p<0.05. Source data are provided as a Source Data file.

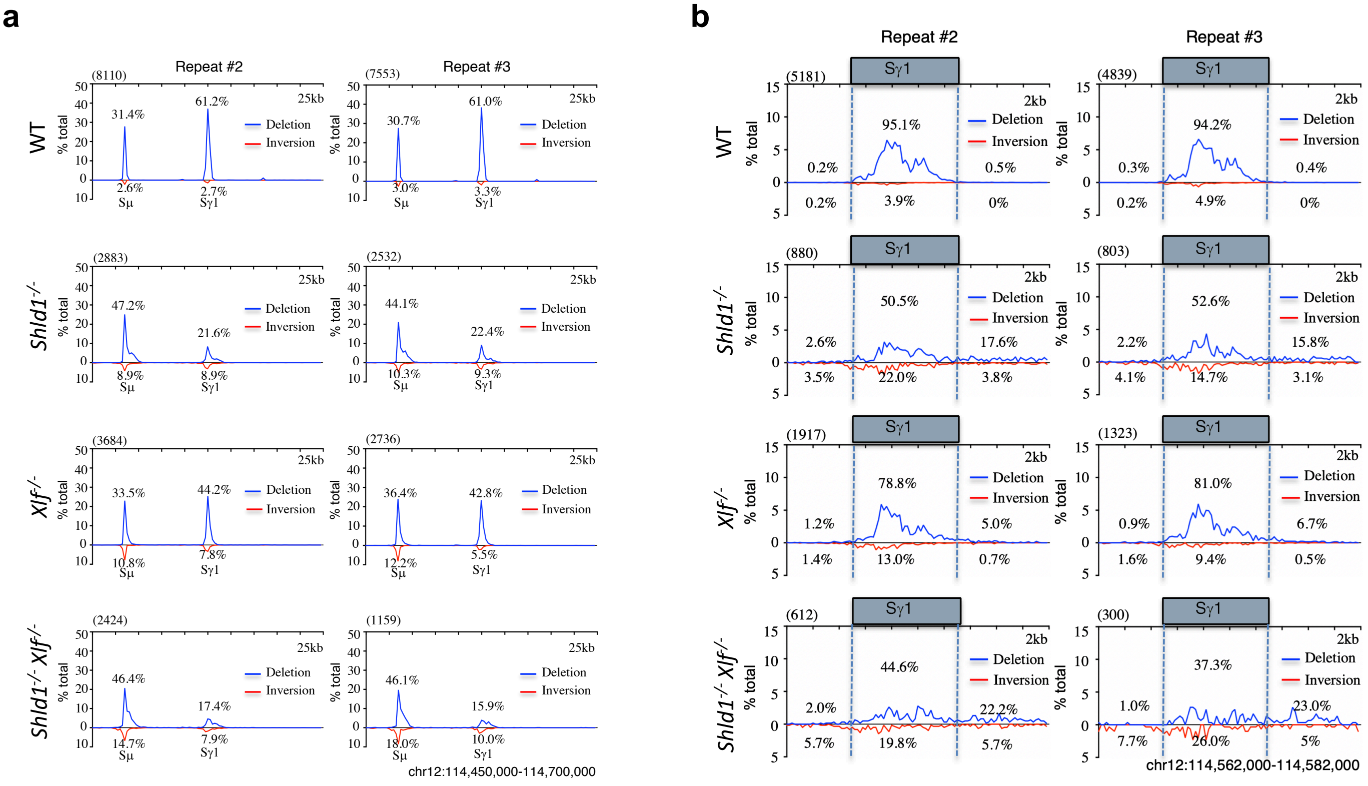

Supplementary Figure 7 (related to Figure 5). CSR-HTGTS-Seq analysis. a CSR-HTGTS-seq analysis of break joining between 5′Sμ and downstream acceptor S regions in WT, *Shld1*$^{-/-}$, *Xlf*$^{-/-}$ *and Shld1*$^{-/-}$*Xlf*$^{-/-}$ splenic B cells stimulated with αIgD dextran/LPS/IL-4. Repeats #2 and #3. **b** Zoom-in view of CSR-HTGTS-Seq junctions located in the AID-targeted ectopic Sγ1 region from WT, *Shld1*$^{-/-}$, *Xlf*$^{-/-}$ *and Shld1*$^{-/-}$*Xlf*$^{-/-}$ cells, repeats #2 and #3. Junctions are plotted at 200 pb bin size. Source data are provided as a Source Data file.

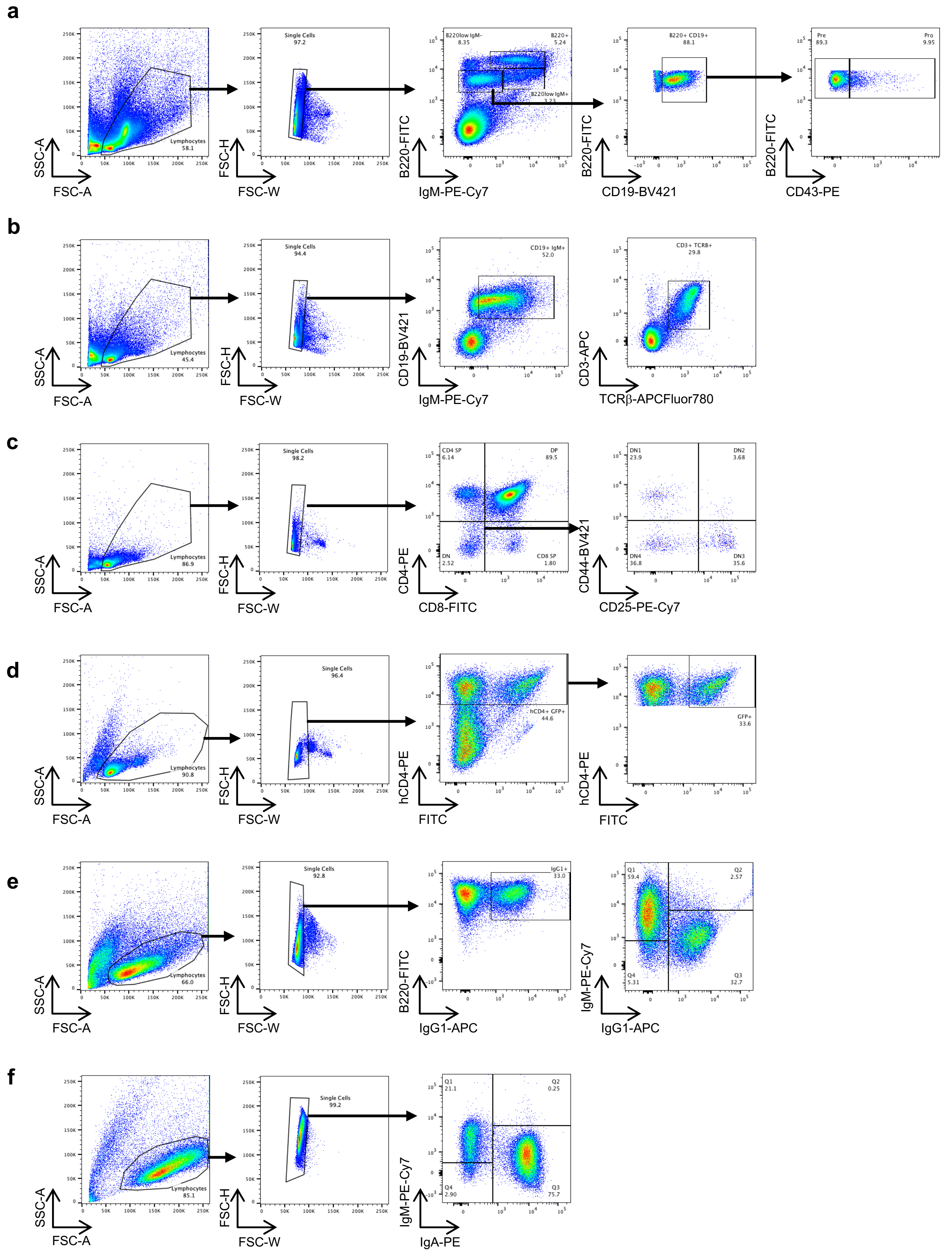

Supplementary Figure 8. Gating strategies. **a** Gating strategy to determine the percentage of CD43⁻B220^{lo}CD19⁺IgM⁻ pre-B cells versus CD43⁺B220^{lo}CD19⁺IgM⁻ pro-B cells in the bone marrow presented on Figure 1e and Supplementary Figure 6c. **b** Gating strategy to determine the percentage of CD19⁺ IgM⁺ and CD3⁺ TCRβ⁺ cells in the spleen presented on Supplementary Figures 1g, j, 6c. **c** Gating strategy to determine the percentage of CD44⁻ CD25⁺ (DN3) and CD44⁻ CD25⁻ (DN4) cells in the thymus presented on Figure 1g and Supplementary Figure 6d. **d** Gating strategy to determine the percentage of hCD4⁺ GFP⁺ pro-B cells presented on Figure 2i and Supplementary Figure 2e. **e** Gating strategy to determine the percentage of switching primary B cells presented on Figures 3b-d. **f** Gating strategy to determine the percentage of switching CH12F3 cells presented on Figure 3j .

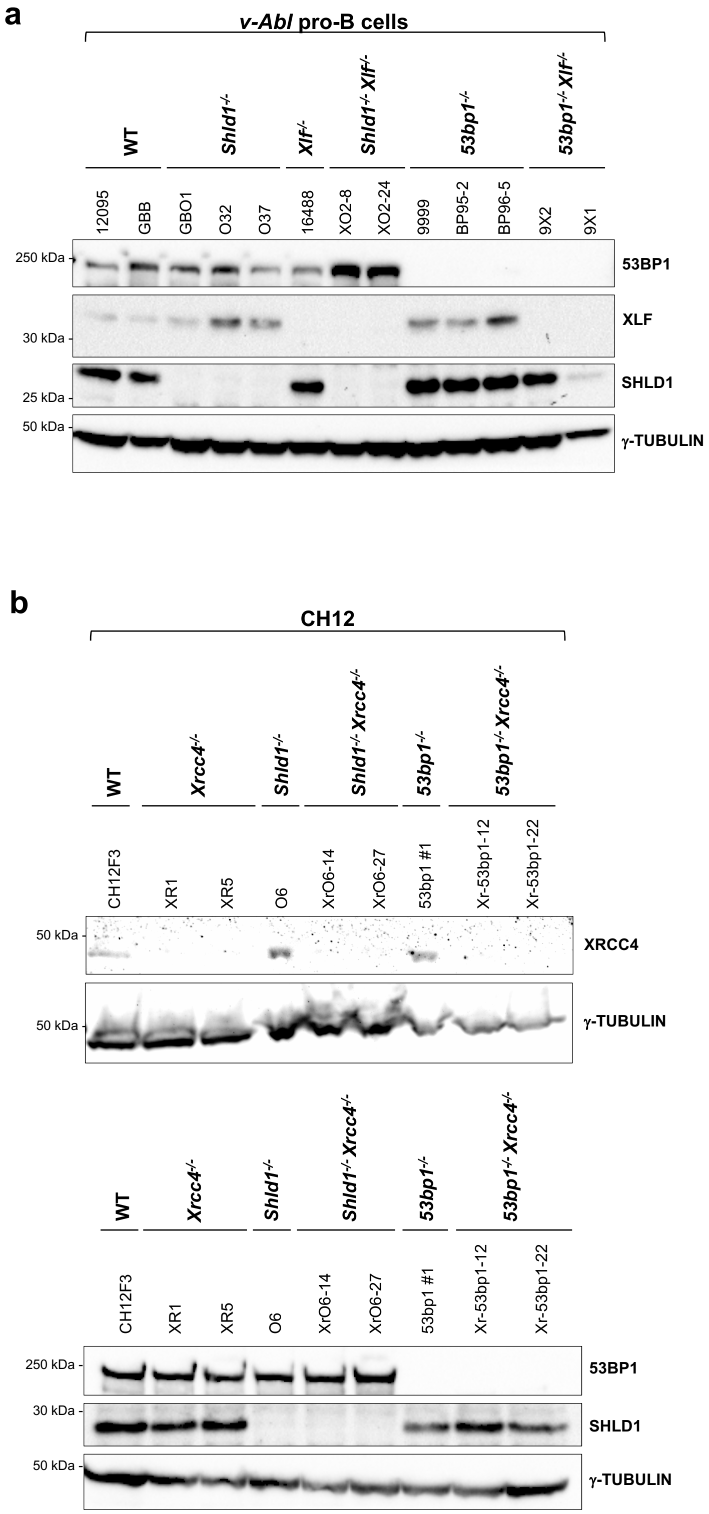

Supplementary Figure 9. Genotyping of *v-abl* ProB cells and CH12 cell lines. a,b Representative Western Blots analysis of **a** *v-abl* ProB cells and **b** CH12 cell lines. Each immunoblot was repeated twice independently with similar results. Source data are provided as a Source Data file.

		% of B220^{low}IgM^{-}CD19^{+} cells		
**Genotype**	B220^{low}IgM^{-} (%)	CD43^{+} (proB)	CD43^{-} (preB)	Pre/ProB ratio
**WT (n=12)**	12.0±3.5	16.9±2.6	82.6±2.6	5.2±1.2
***Shld1*$^{-/-}$ (n=10)**	10.2±1.6	17.5±3.4	82.0±3.4	5.2±1.6
***53bp1*$^{-/-}$ (n=4)**	5.2±1.2	29.0±3.7	69.8±3.6	2.5±0.4
***Xlf*$^{-/-}$ (n=11)**	7.7±2.2	23.6±3.6	76.0±3.6	3.4±0.6
***Shld1*$^{-/-}$ *Xlf*$^{-/-}$ (n=11)**	8.4±2.1	17.0±2.5	82.7±2.5	5.0±0.8

Supplementary Table 1. Analysis of B cell development in the bone marrow of *Shld1*$^{-/-}$ and *Shld1*$^{-/-}$ *Xlf*$^{-/-}$ mice.

	Cells numbers (x10^6)				
**Genotype**	Total	CD19$^+$IgM$^+$	CD3$^+$TCRβ$^+$	CD4 SP	CD8 SP
**WT (n=13)**	149.9±24.4	81.3±15.3	46.3±7.8	27.7±4.4	19.7±3.8
***Shld1$^{-/-}$* (n=10)**	143.8±15.6	75.2±8.1	46.9±5.7	27.2±2.8	21.2±2.7
***53bp1$^{-/-}$* (n=4)**	58.8±6.7	27.5±4.7	16.3±1.3	9.0±0.8	8.4±0.6
***Xlf$^{-/-}$* (n=11)**	81.3±13.9	36.0±5.4	28.7±5.6	17.7±3.6	12.2±2.5
***Shld1$^{-/-}$Xlf$^{-/-}$* (n=11)**	77.7±14.2	35.1±6.0	25.8±5.3	16.1±3.2	10.9±2.1

Supplementary Table 2. Analysis of B and T cell development in the spleen of *Shld1$^{-/-}$* and *Shld1$^{-/-}$ Xlf$^{-/-}$* mice.

	Cell numbers (x10^6)					% of DN cells	
**Genotype**	Total	DP	CD4 SP	CD8 SP	DN	DN3	DN4
**WT (n=10)**	100.6±20.8	86.3±17.1	8.4±2.5	2.8±0.9	3.0±0.7	36.8±2.2	42.6±6.9
***Shld1$^{-/-}$* (n=10)**	113.5±32.0	97.4±28.9	9.3±3.0	3.0±1.1	3.8±1.2	35.7±2.9	47.4±8.5
***53bp1$^{-/-}$* (n=4)**	41.6±22.8	36.0±20.9	2.4±0.6	1.0±0.2	2.1±1.0	54.3±6.8	15.7±3.1
***Xlf$^{-/-}$* (n=8)**	49.7±22.9	40.1±20.6	4.6±1.4	1.9±0.7	3.1±1.3	49.1±2.7	31.8±1.7
***Shld1$^{-/-}$Xlf$^{-/-}$* (n=9)**	57.6±15.0	48.6±12.3	3.8±1.3	1.8±0.6	3.4±1.0	50.3±4.3	34.5±4.2

Supplementary Table 3. Analysis of T cell development in the thymus of *Shld1$^{-/-}$* and *Shld1$^{-/-}$ Xlf$^{-/-}$* mice.

Genotype		Metaphases with Igh locus breaks	Metaphases with Igh locus translocations	Total aberrant metaphases	Total normal metaphases	Total metaphases analyzed	% aberrant metaphases	% IgH breaks	% IgH translocations
**WT**	Exp #1	3	0	3	197	200	1.5	1.5	0
		3	1	4	196	200	2	1.5	0.5
	Exp #2	2	1	3	197	200	1.5	1	0.5
	Exp #3	3	1	4	201	205	2	1.5	0.5
	Exp #4	1	1	2	198	200	1	0.5	0.5
	TOTAL	12	4	16	989	1005	1.6	1.2	0.4
Shld1$^{-/-}$	Exp #1	15	1	16	184	200	8	7.5	0.5
	Exp #2	15	2	17	183	200	8.5	7.5	1
		21	1	22	243	265	8.3	7.9	0.4
	Exp #3	26	1	27	183	210	12.9	12.4	0.5
	Exp #4	24	1	25	175	200	12.5	12	0.5
	TOTAL	101	6	107	968	1075	10	9.4	0.6
Xlf$^{-/-}$	Exp #1	7	1	8	183	191	4.2	3.7	0.5
		4	0	4	196	200	2	2	0
	Exp #2	6	1	7	193	200	3.5	3	0.5
		4	0	4	196	200	2	2	0
	Exp #3	13	1	14	196	210	6.7	6.2	0.5
	Exp #4	15	2	17	183	200	8.5	7.5	1.0
	TOTAL	49	5	54	1147	1201	4.5	4.1	0.4
Shld1$^{-/-}$ Xlf$^{-/-}$	Exp #1	16	1	17	183	200	8.5	8	0.5
		15	0	15	175	190	7.9	7.9	0
	Exp #2	13	1	14	186	200	7	6.5	0.5
	Exp #3	22	1	23	187	210	11	10.5	0.5
	Exp #4	22	1	23	177	200	11.5	11	0.5
	TOTAL	88	4	92	908	1000	9.2	8.8	0.4

Supplementary Table 4. Genomic instability at the *Igh* locus in primary B cells undergoing CSR. Number and percentage of aberrant metaphases harboring chromosome breaks and/or translocations involving the Igh locus from stimulated B cells of the indicated genotype.

Two-sided Fischer's exact test	p value	
WT vs *Shld1$^{-/-}$*	2.3280401656265E-17	****
WT vs *Xlf$^{-/-}$*	7.9179414896933E-5	****
WT vs *Shld1$^{-/-}$Xlf$^{-/-}$*	4.2129398599184E-15	****
Xlf$^{-/-}$ vs *Shld1$^{-/-}$Xlf$^{-/-}$*	1.4254772216385E-5	****
Shld1$^{-/-}$ vs *Shld1$^{-/-}$Xlf$^{-/-}$*	0.60166146775667	ns
Xlf$^{-/-}$ vs *Shld1$^{-/-}$*	4.4689100840892E-7	****

Genotype		Metaphases with Igh locus breaks	Metaphases with Igh locus translocations	Total aberrant metaphases	Total normal metaphases	Total metaphases analyzed	% aberrant metaphases	% IgH breaks	% IgH translocations
**WT**	Exp #1	3	0	3	197	200	1.5	1.5	0
	Exp #2	3	0	3	197	200	1.5	1.5	0
	Exp #3	5	0	5	195	200	2.5	2.5	0
	Exp #4	2	0	2	198	200	1	1	0
	TOTAL	13	0	13	787	800	1.6	1.6	0
Shld1^-/-	Exp #1	21	1	22	178	200	11	10.5	0.5
	Exp #2	32	0	32	168	200	16	16.0	0
	Exp #3	26	3	29	171	200	14.5	13	1.5
	Exp #4	9	1	10	190	200	5	4.5	0.5
	TOTAL	88	5	93	707	800	11.6	11	0.6
Xrcc4^-/-	Exp #1	7	0	7	193	200	3.5	3.5	0
	Exp #2	13	0	13	187	200	6.5	6.5	0
	Exp #3	11	0	11	189	200	5.5	5.5	0
	Exp #4	8	1	9	191	200	4.5	4	0.5
	TOTAL	39	1	40	760	800	5	4.9	0.1
Shld1^-/- Xrcc4^-/-	Exp #1	25	2	27	173	200	13.5	12.5	1
	Exp #2	23	2	25	175	200	12.5	11.5	1
	Exp #3	20	1	21	179	200	10.5	10	0.5
	TOTAL	68	5	73	527	600	12.2	11.3	0.8

Supplementary Table 5. Genomic instability at the Igh locus in primary B cells undergoing CSR. Number and percentage of aberrant metaphases harboring chromosome breaks and/or translocations involving the Igh locus from stimulated B cells of the indicated genotype.

Two-sided Fischer's exact test	p value	
WT vs *Shld1$^{-/-}$*	5.43550432286E-17	****
WT vs *Shld1$^{-/-}$Xrcc4$^{-/-}$*	1.3654665051398E-16	****
Shld1$^{-/-}$ vs *Shld1$^{-/-}$Xrcc4$^{-/-}$*	1	ns
Xrcc4$^{-/-}$ vs *Shld1$^{-/-}$Xrcc4$^{-/-}$*	1.4564541562486E-6	****

#replicate	count	mean	std	min	25 %	50 %	75 %	max	% reads smaller than 7,160 bp
**WT #1**	342	8988.833333	1341.31854581563	4687	8119.5	9105.5	9896	12939	9.94152046783625
**WT #2**	673	8517.240713	1931.01468400115	303	7734	8718	9704	14100	16.3447251114413
**WT #3**	261	8423.804598	1965.96184429137	500	7416	8510	9642	13163	21.0727969348659
**WT**	1276	8624.52742946708	1812.33771515047	303	7746	8810.5	9763.5	14100	15.5956112852664
Shld1$^{-/-}$ **#1**	849	5949.09305064782	3358.07770175158	1359	2794	5892	8444	13931	63.3686690223792
Shld1$^{-/-}$ **#2**	795	5103.04150943396	3241.16394213346	426	2783.5	4157	7610.5	13297	71.0691823899371
Shld1$^{-/-}$ **#3**	1542	5412.59533073929	3359.6146392111	436	2407	4675	8398	13635	65.5642023346303
Shld1$^{-/-}$	3186	5478.31763967357	3343.43859311307	426	2658.5	4679	8305	13931	66.3527934714375
Xlf$^{-/-}$ **#1**	616	7759.42694805194	2718.23925755651	2065	5292	8596	9845.5	13261	36.038961038961
Xlf$^{-/-}$ **#2**	1222	7036.74631751227	3003.15426333181	244	4563.5	7498.5	9245.75	13965	45.4173486088379
Xlf$^{-/-}$ **#3**	865	6517.62774566473	2865.62102662071	2263	4327	6392	8883	14405	56.4161849710982
Xlf$^{-/-}$	2703	7035.31594524602	2930.60900218363	244	4606	7456	9359.5	14405	46.7998520162782
Shld1$^{-/-}$ ***Xlf***$^{-/-}$ **#1**	2589	3962.09926612591	2455.88412558241	1229	2311	3169	4793	14021	87.369640787949
Shld1$^{-/-}$ ***Xlf***$^{-/-}$ **#2**	1893	4636.26994189117	2698.3486357341	436	2554	3947	6311	15733	82.6201796090861
Shld1$^{-/-}$ ***Xlf***$^{-/-}$ **#3**	1716	3990.64044289044	2656.64951829614	1149	2018	3017	4873	12891	85.2564102564102
Shld1$^{-/-}$ ***Xlf***$^{-/-}$	6198	4175.9072281381	2605.45035455473	436	2312	3288	4949	15733	85.3339787028073

Supplementary Table 6. Long-read high-throughput sequencing analysis. Number of reads, mean read length, standard deviation, reads distribution and percentage of reads smaller than 7,160 bp for each genotype. n = 3 for each genotype.

Primers	Sequence (5' - 3’)	Reference
TCRB-D1U-S	GAGGAGCAGCTTATCTGGTGGTTT	Gartner F. et al., Immunity. 1999
TCRB-D2US	GTAGGCACCTGTGGGGAAGAAACT	Aifantis I. et al., Immunity. 1997
TCRB-J1D-A	CACAACCCCTCCAGTCAGAAATG	Gartner F. et al., Immunity. 1999
TCRB-J2D-A	TGAGAGCTGTCTCCTACTATCGATT	Aifantis I. et al., Immunity. 1997
IMR42	CTAGGCCACAGAATTGAAAGATCT	Bredemeyer, A. L. et al., Nature 2006
IMR43	GTAGGTGGAAATTCTAGCATGATGC	Bredemeyer, A. L. et al., Nature 2006
pkja2	GCCACAGACATA GACAACGGAA	Lescale C. et al., Nat Com. 2016
pk6c	GTTGCTGTGGTTGTCTGGTG	Bredemeyer, A. L. et al., Nature 2006
pk6d	GAAATACATCAGACCAGCATGG	Bredemeyer, A. L. et al., Nature 2006
5µ3	AATGGATACCTCAGTGGTTTTTAATGGTGGGTTTA	Reina-San-Martin B. et al., J Exp Med. 2003
γ1-R	CAATTAGCTCCTGCTCTTCTGTGG	Reina-San-Martin B. et al., J Exp Med. 2003
EµF	GCCGATCAGAACCAGAACAC	Ling AK. et al., EMBO. 2020
Cg1R2	GGAAAGCGAGATGAGCAGAC	This study
Hprt forward	CTGGTGAAAAGGACCTCTCG	Xu, G. et al. Nature 2015
Hprt reverse	TGAAGTACTCATTATAGTCAAGGGCA	Xu, G. et al. Nature 2015
Aid forward	GAAAGTCACGCTGGAGACCG	Xu, G. et al. Nature 2015
Aid reverse	TCTCATGCCGTCCCTTGG	Xu, G. et al. Nature 2015
Ig1-Fw	GGCCCTTCCAGATCTTTGAG	Nguyen HV. Et al., PNAS. 2017
Cg1-Rv	GGATCCAGAGTTCCAGGTCACT	Nguyen HV. Et al., PNAS. 2017
Ig3-Fw	TGGGCAAGTGGATCTGAACA	Nguyen HV. Et al., PNAS. 2017
Cg3-Rv	CTCAGGGAAGTAGCCTTTGACA	Nguyen HV. Et al., PNAS. 2017
DF1	CAGACACCGATTCACATGCT	This study
DR1	TCCTTATTCCCACGAAGCAC	This study
EF1	CCGAGTTGTAGGTGAAGACCA	This study
ER1	CGAGAAAAGGTCAAGGCAAG	This study
Shld1-Fw	ACCTTTACCCACTGAGCCTC	Dev H. et al., Nat Cell Biol. 2018
Shld1-Rv	GTAAAAGCGTGTGTCCCTGG	Dev H. et al., Nat Cell Biol. 2018
Xrcc4-Fw	CCCTCACAGAAACACAACTCA	Yu W. et al. Nat Com. 2020
Xrcc4-Rv	CAAGGAGGTGGCCACTAGTT	Yu W. et al. Nat Com. 2020
Xlf-Fw	CTATGGAAGCCAGGAGAGAATG	Vera, G. et al. Mol. Cell. Biol. 2012
Xlf-Rv	GTCCCCAGCTGTTAAGAGTTTC	Vera, G. et al. Mol. Cell. Biol. 2012
Off-SHLD1-sgRNA1-F	ACTGCATGAGCTCACCATCA	This study
Off-SHLD1-sgRNA1-R	GCAGTGATGCAAGCTTTCTG	This study
Off-SHLD1-sgRNA2-F	AATCCAGGATCTGTGGTTGC	This study
Off-SHLD1-sgRNA2-R	GTCAAGTCACGAGTCATCCAT	This study
Off-SHLD1-sgRNA3-F	CTTATGCTGCGTGGGGTTAT	This study
Off-SHLD1-sgRNA3-R	TCTGCATCTAAGGGCAGCAT	This study
Off-SHLD1-sgRNA4-F	ATGCTGCAACTCCATGTACC	This study
Off-SHLD1-sgRNA4-R	CCCCTAGCCTTTATTCCTTCC	This study
EV-5Dd1SE	TGTGCACCTGAGTTTTAGGACTCT	This study
EV-3Dd2R	CAGGAGGCCTGGGAGACGGTTCTTCACCCT	This study
EV-5Dd2	TGTGGCTTGACATGCAGAAAACACCTG	This study
EV-3Jd1C	CTTGGTTCCACAGTCACTTGGGTT	This study
EV-3Jd1F3	CCTCTTCCCCCTCCAAAGAAA	This study
EV-3Jd1R6	CCCCAAGCCGCTTCTTAGCTTTTC	This study
DV104F2	ACTCGCAGCTGGGGGATGCT	Difilippantinio S et al, Nature 2008
DV105F3	TGTTCAAGGTCGATTTTCTGTG	Difilippantinio S et al, Nature 2008
sgRNAs	Sequence (5' - 3’)	
Shld1 sgRNA-1	CCAACTGGCCTTTCGAAGA	Dev H. et al., Nat Cell Biol. 2018
Shld1 sgRNA-2	GATGGACTTCGGAAATCCC	Dev H. et al., Nat Cell Biol. 2018
Xrcc4 sgRNA-1	GAATGTATAACAGGAGACGG	Lenden H. et al. JIM 2017
Xrcc4 sgRNA-2	CTTTTCTAAGGAGTCTCGGC	Lenden H. et al. JIM 2017
Xlf sgRNA-1	TTAGCATACACCAACTTC	Lenden H. et al. JIM 2017
Xlf sgRNA-2	CACCAACAGGTACTCATA	Lenden H. et al. JIM 2017
53bp1 sgRNA1	GCGACCTGGGGATCGACC	This study
53bp1 sgRNA2	CGAGCGGAGCCGAGCTAG	This study

Supplementary Table 7. List of primers and gRNAs sequences

	Genotype	Clone ID	Allele 1	Allele 2	Generation method	Origin	Reference
v-Abl pro-B cells	**WT**	12095	NA	NA	v-Abl/Bcl2 immortalization of bone marrow pro-B cells	Mouse	Lescale C. et al., Nat Com. 2016
		12096	NA	NA	v-Abl/Bcl2 immortalization of bone marrow pro-B cells	Mouse	Lescale C. et al., Nat Com. 2016
		GBB	NA	NA	v-Abl/Bcl2 immortalization of bone marrow pro-B cells	Mouse	This study
		O38	NA	NA	CRISPR-Cas9 genome editing	GBB	This study
	53bp1$^{-/-}$	9999	NA	NA	v-Abl/Bcl2 immortalization of bone marrow pro-B cells	Mouse	Liu X. et al., PNAS. 2012
		1110	NA	NA	v-Abl/Bcl2 immortalization of bone marrow pro-B cells	Mouse	Liu X. et al., PNAS. 2012
		BP95-2*	ND	ND	CRISPR-Cas9 genome editing	12095	This study
		BP96-5*	ND	ND	CRISPR-Cas9 genome editing	12095	This study
	Shld1$^{-/-}$	O32	-2bps		CRISPR-Cas9 genome editing	GBB	This study
		O37	-10bps		CRISPR-Cas9 genome editing	GBB	This study
		O44	-17bps		CRISPR-Cas9 genome editing	GBB	This study
		GBO1	NA	NA	v-Abl/Bcl2 immortalization of bone marrow pro-B cells	Mouse	This study
	Xlf$^{-/-}$	16488	NA	NA	v-Abl/Bcl2 immortalization of bone marrow pro-B cells	Mouse	Lescale C. et al., Cell Rep. 2016
		X95-3	-315bps	-317bps	CRISPR-Cas9 genome editing	12095	Lescale C. et al., Cell Rep. 2016
	Shld1$^{-/-}$ ***Xlf***$^{-/-}$	XO2-8	-82bps	-62bps	CRISPR-Cas9 genome editing	16488	This study
		XO2-24	-82bps		CRISPR-Cas9 genome editing	16488	This study
	53bp1$^{-/-}$ ***Xlf***$^{-/-}$	9x1	-317bps	-403bps	CRISPR-Cas9 genome editing	9999	This study
		9x2	inversion		CRISPR-Cas9 genome editing	9999	This study
CH12	**WT**	CH12	NA	NA		Mouse	Nakamura M. et al., International Immunology 1996
		O2	NA	NA		CH12-Cas9	Dev H. et al., Nat Cell Biol. 2018
	53bp1$^{-/-}$	53bp1 #1	ND	ND	CRISPR-Cas9 genome editing		Panchakshari R.A. et al. PNAS 2018
		53bp1 #2	ND	ND	CRISPR-Cas9 genome editing		Panchakshari R.A. et al. PNAS 2018
	Shld1$^{-/-}$	O6	-17bps		CRISPR-Cas9 genome editing	CH12-Cas9	Dev H. et al., Nat Cell Biol. 2018
		O12	-25bps	-186bps	CRISPR-Cas9 genome editing	CH12-Cas9	Dev H. et al., Nat Cell Biol. 2018
	Xrcc4$^{-/-}$	XR5	-288bps	-289bps	CRISPR-Cas9 genome editing	CH12-Cas9	This study
		XR1	-336bps	-599bps	CRISPR-Cas9 genome editing	CH12-Cas9	This study
	Shld1$^{-/-}$ ***Xrcc4***$^{-/-}$	XRO6-14	inversion	-306bps	CRISPR-Cas9 genome editing	O6	This study
		XRO6-27	-301bps		CRISPR-Cas9 genome editing	O6	This study
	53bp1$^{-/-}$ ***Xrcc4***$^{-/-}$	XR-53BP1-12	-289bps	-289bps	CRISPR-Cas9 genome editing	53bp1 #1	This study
		XR-53BP1-22	-775bps	-980bps	CRISPR-Cas9 genome editing	53bp1 #1	This study

* Validation by WB

Supplementary Table 8. List of cell lines used in the study

Antibody	Catalogue number	Dilution
anti-SHLD1	Thermo Fisher Scientific, PA5-559280	1:200
anti-XLF	Bethyl Laboratories, A300-729A	1:1000
anti-γ-TUBULIN	Sigma Aldrich, clone GTU-88, T6557	1:10000
anti-MAD2L2	Protein Tech, 12683-1-AP	1:500
anti-FLAG	Protein Tech, 20543-1-AP	1:2000
anti-LAMIN B	Abcam, 16048	1:2000
anti-53BP1	Novus Biologicals, NB100-304	1:1000
anti-XRCC4	Santa Cruz, clone C20, sc-8285	1:1300
HRP-linked anti-Mouse IgG	Cell Signaling Technology, 7076	1:10000
HRP-linked anti-Rabbit IgG	Cell Signaling Technology, 7074	1:5000
LI-COR IRDye® 800CW Donkey anti-Goat IgG	LI-COR, 926-32214	1:10000
LI-COR IRDye® 680RD Goat anti-Mouse IgG	LI-COR, 926-68070	1:20000
anti-hCD4	Miltenyi, clone M-T466, 130-113-254	1:100
anti-CD16-32	BD Biosciences, clone 2.4G2, 553142	1:200
anti-CD19	BD Biosciences, clone 1D3, 560375	1:200
anti-CD43	BD Biosciences, clone S7, 553271	1:150
anti-B220	BD Biosciences, clone RA3–6B2, 557669	1:200
anti-B220	BD Biosciences, clone RA3–6B2, 553092	1:200
anti-IgM	BD Biosciences, clone R6–60.2, 552867	1:200
anti-CD4	BD Biosciences, clone RM4–5, 553048	1:200
anti-CD8	BD Biosciences, clone 53-6.7, 557668	1:200
anti-CD3e	BD Biosciences, clone 145-2C11, 553066	1:200
anti-CD44	BD Biosciences, clone IM7, 560451	1:200
anti-CD25	BD Biosciences, clone PC61, 552880	1:200
anti-TCRβ	eBioscience, clone H57–597, 47-5961-82	1:200
anti-CD93	BD Biosciences, clone AA4.1, 558039	1:200
anti-CD23	BD Biosciences, clone B3B4, 562929	1:200
anti-CD21	BD Biosciences, clone 7G6, 561770	1:500
anti-IgG1	BD Biosciences, clone X56, 550874	1:500
anti-IgG2b	Biolegend, clone RMG2b-1, 406708	1:500
anti-IgG3	BD Biosciences, clone R40-82, 553403	1:500
anti-Thy1.1	BD Bioscience, clone HIS51, 740044	1:300
anti-IgA	eBiosciences, clone mA-6E1, 12-4204-82	1:200
Goat anti-mouse IgGs	Jackson ImmunoResearch	250 ng
Goat anti-mouse IgMs	Jackson ImmunoResearch	250 ng
Goat anti-mouse IgG1 antibodies	Southern Biotech	250 ng
Purified mouse IgG, IgG1	Sigma-Aldrich	Starting at 12 µg/ml and seven consecutive 1:3 dilutions
Purified mouse IgM	Merck Millipore	Starting at 12 µg/ml and seven consecutive 1:3 dilutions

Supplementary Table 9. List of antibodies used in the study
