# Supplementary material for: SHLD1 is dispensable for 53BP1-dependent V(D)J recombination but critical for productive class switch recombination
Source: Nat Commun. 2022 Jun 28;13:3707. doi: 10.1038/s41467-022-31287-3 (PMC9240092; doi:10.1038/s41467-022-31287-3)
Supplement: Supplementary file 2 — Reporting Summary [file 41467_2022_31287_MOESM2_ESM.pdf]

nature portfolio

Corresponding author(s): Ludovic Deriano and Chloé Lescale

Last updated by author(s): May 20, 2022

Reporting Summary

Nature Portfolio wishes to improve the reproducibility of the work that we publish. This form provides structure for consistency and transparency in reporting. For further information on Nature Portfolio policies, see our Editorial Policies and the Editorial Policy Checklist.

Statistics

For all statistical analyses, confirm that the following items are present in the figure legend, table legend, main text, or Methods section.

n/a	Confirmed	
☐	☒	The exact sample size (*n*) for each experimental group/condition, given as a discrete number and unit of measurement
☐	☒	A statement on whether measurements were taken from distinct samples or whether the same sample was measured repeatedly
☐	☒	The statistical test(s) used AND whether they are one- or two-sided<br>*Only common tests should be described solely by name; describe more complex techniques in the Methods section.*
☒	☐	A description of all covariates tested
☒	☐	A description of any assumptions or corrections, such as tests of normality and adjustment for multiple comparisons
☐	☒	A full description of the statistical parameters including central tendency (e.g. means) or other basic estimates (e.g. regression coefficient) AND variation (e.g. standard deviation) or associated estimates of uncertainty (e.g. confidence intervals)
☐	☒	For null hypothesis testing, the test statistic (e.g. *F*, *t*, *r*) with confidence intervals, effect sizes, degrees of freedom and *P* value noted<br>*Give P values as exact values whenever suitable.*
☒	☐	For Bayesian analysis, information on the choice of priors and Markov chain Monte Carlo settings
☒	☐	For hierarchical and complex designs, identification of the appropriate level for tests and full reporting of outcomes
☒	☐	Estimates of effect sizes (e.g. Cohen's *d*, Pearson's *r*), indicating how they were calculated

Our web collection on statistics for biologists contains articles on many of the points above.

Software and code

Policy information about availability of computer code

Data collection	Gel/membrane Imaging: Image Lab v6.0;<br>Microscopy: Metafer 4 v3.11.3;<br>Flow cytometry: FACS Diva v8.0.1;<br>ELISA: Magellan software v7.2(TECAN);<br>CSR-HTGTS : Next-Seq™550 (Illumina);<br>Pacbio : long-read PacBio Sequel I technology;<br>Real-time PCR : QuantStudio Design and Analysis Software v2.6.0
Data analysis	Software used:<br>Flowjo v10.4.2;<br>Graphpad Prism v7.04;<br>Image Lab v6.0;<br>R v3.5.1;<br>Microsoft Excel v16.16.2;<br>QuantStudio Design and Analysis Software v2.6.0;<br>SMRTLINK software from PacBio (v9) (smrtlink: https://www.pacb.com/support/softwaredownloads/);<br>Minimap2;<br>Sequana library (Thomas Cokelaer, Desvillechabrol, D., Legendre, R. & Cardon, M. 'Sequana': a Set of Snakemake NGS pipelines. The JOurnal of Open Source Software (2017));<br>HTGTS libraries were processed via published pipeline (Hu, J. et al. Detecting DNA double-stranded breaks in mammalian genomes by linear amplification-mediated high-throughput genome-wide translocation sequencing. Nat Protoc 11, 853-871 (2016)).

Statistical analysis were perfomed using BiostaTGV (https://biostatgv.sentiweb.fr) and Graphpad Prism v7.04.

For manuscripts utilizing custom algorithms or software that are central to the research but not yet described in published literature, software must be made available to editors and reviewers. We strongly encourage code deposition in a community repository (e.g. GitHub). See the Nature Portfolio guidelines for submitting code & software for further information.

Data

Policy information about availability of data

All manuscripts must include a data availability statement. This statement should provide the following information, where applicable:

- Accession codes, unique identifiers, or web links for publicly available datasets
- A description of any restrictions on data availability
- For clinical datasets or third party data, please ensure that the statement adheres to our policy

The CSR-HTGTS data have been deposited in NCBI's Gene Expression Omnibus under the accession number GSE202567. The Long range PCR sequencing (Pacbio) data have been deposited in NCBI's Sequence Read Archive under the accession number PRJNA831666. All other data can be found in the Supplementary Data of this paper or in the Source Data. This includes all uncropped blots, gels, and data shown in graphs throughout the manuscript, including the Supplementary Figures. All data are available from the authors upon reasonable request.

Field-specific reporting

Please select the one below that is the best fit for your research. If you are not sure, read the appropriate sections before making your selection.

[x] Life sciences [ ] Behavioural & social sciences [ ] Ecological, evolutionary & environmental sciences

For a reference copy of the document with all sections, see nature.com/documents/nr-reporting-summary-flat.pdf

Life sciences study design

All studies must disclose on these points even when the disclosure is negative.

Sample size	No statistical methods were used to predetermine sample size for all experiments. Sample sizes were chosen based on previous studies in this field that used similar sample sizes to generate reproducible results (Lescale C et al, Nat Com, 2016; Dev H et al, Nature Cell Biology, 2018). The number of independent experiments are indicated in the legend of each Figure.
Data exclusions	No data was excluded from this study.
Replication	All experiments were repeated by at least two biological replicates with two or three isogenetic clones with consistent results. All attempts at replication were successful.
Randomization	Sex- and age-matched mice were used. Randomization is not relevant because we did not use different experimental groups in our study.
Blinding	No experiments were blinded, since subjective rating of data was not involved. Investigators were not blinded to allocation during experiments and outcome assessment. Blinding was not possible as investigators need to verify the control and matched mutant mouse strains as well as cell lines before each experiment. For many other approaches used in the manuscript, including Western blots, Flow Cytometry and IP, blinding was not feasible.

Reporting for specific materials, systems and methods

We require information from authors about some types of materials, experimental systems and methods used in many studies. Here, indicate whether each material, system or method listed is relevant to your study. If you are not sure if a list item applies to your research, read the appropriate section before selecting a response.

Materials & experimental systems

n/a	Involved in the study	
[ ]	[x]	Antibodies
[ ]	[x]	Eukaryotic cell lines
[x]	[ ]	Palaeontology and archaeology
[ ]	[x]	Animals and other organisms
[x]	[ ]	Human research participants
[x]	[ ]	Clinical data
[x]	[ ]	Dual use research of concern

Methods

n/a	Involved in the study	
[x]	[ ]	ChIP-seq
[ ]	[x]	Flow cytometry
[x]	[ ]	MRI-based neuroimaging

Antibodies

Antibodies used

Primary antibodies used in Western blot studies:
anti-SHLD1: Thermo Fisher Scientific, PA5-559280, 1:200
anti-XLF: Bethyl Laboratories, A300-729A, 1:1000
anti-γ-TUBULIN: Sigma Aldrich, clone GTU-88, T6557, 1:10000
anti-MAD2L2: Protein Tech, 12683-1-AP, 1:500
anti-FLAG: Protein Tech, 20543-1-AP, 1:2000
anti-LAMIN B : Abcam, 16048, 1:2000
anti-53BP1 : Novus Biologicals, NB100-304, 1:1000
anti-XRCC4 : Santa Cruz, clone C20, sc-8285, 1:1300

Secondary anti-IgG antibodies used in Western blot studies:
HRP-linked anti-Mouse IgG: Cell Signaling Technology, 7076, 1:10000
HRP-linked anti-Rabbit IgG: Cell Signaling Technology, 7074, 1:5000
LI-COR IRDye® 800CW Donkey anti-Goat IgG, LI-COR, 926-32214, 1:10,000
LI-COR IRDye® 680RD Goat anti-Mouse IgG, LI-COR, 926-68070, 1:20,000

Antibodies used in Flow Cytometry studies:
anti-hCD4: Miltenyi, clone M-T466, 130-113-254, 1:100
anti-CD16-32: BD Biosciences, clone 2.4G2, 553142, 1:200
anti-CD19: BD Biosciences, clone 1D3, 560375, 1:200
anti-CD43: BD Biosciences, clone S7, 553271, 1:150
anti-B220: BD Biosciences, clone RA3–6B2, 557669, 1:200
anti-B220: BD Biosciences, clone RA3–6B2, 553092, 1:200
anti-IgM: BD Biosciences, clone R6–60.2, 552867, 1:200
anti-CD4: BD Biosciences, clone RM4–5, 553048, 1:200
anti-CD8: BD Biosciences, clone 53-6.7, 557668, 1:200
anti-CD3e: BD Biosciences, clone 145-2C11, 553066, 1:200
anti-CD44: BD Biosciences, clone IM7, 560451, 1:200
anti-CD25: BD Biosciences, clone PC61, 552880, 1:200
anti-TCRβ: eBioscience, clone H57–597, 47-5961-82, 1:200
anti-CD93 : BD Biosciences, clone AA4.1, 558039, 1:200
anti-CD23 : BD Biosciences, clone B3B4, 562929, 1:200
anti-CD21 : BD Biosciences, clone 7G6, 561770, 1:500
anti-IgG1: BD Biosciences, clone X56, 550874, 1:500
anti-IgG2b: Biolegend, clone RMG2b-1, 406708, 1:500
anti-IgG3: BD Biosciences, clone R40-82, 553403, 1:500
anti-Thy1.1: BD Bioscience, clone HIS51, 740044, 1:300
anti-IgA: eBiosciences, clone mA-6E1, 12-4204-82, 1:200

Antibodies used in ELISA studies:
Goat anti-mouse IgGs, anti-mouse IgMs (Jackson ImmunoResearch), or anti-mouse IgG1 antibodies (Southern Biotech) 250 ng. Purified mouse IgG, IgG1 (Sigma-Aldrich) and IgM (Merck Millipore) antibodies starting at 12 μg/ml and seven consecutive 1:3 dilutions.

Validation

Primary antibodies used in Western blot studies:
anti-SHLD1: Thermo Fisher Scientific, PA5-559280, validated in WB (0.04-0.4 μg/mL) (https://www.thermofisher.com/antibody/product/C20orf196-Antibody-Polyclonal/PA5-59280)
anti-XLF: Bethyl Laboratories, A300-729A, (https://www.ncbi.nlm.nih.gov/pmc/articles/PMC5323033/)
anti-γ-TUBULIN: Sigma Aldrich, clone GTU-88, T6557, validated in WB (1:10,000) (https://www.sigmaaldrich.com/FR/fr/product/sigma/t6557)
anti-MAD2L2: Protein Tech, 12683-1-AP, validated in WB (1:500 - 1:2,400) (https://www.ptglab.com/products/MAD2L2-Antibody-12683-1-AP.htm)
anti-FLAG: Protein Tech, 20543-1-AP, validated in WB (1:2,000-1:10,000) (https://www.ptglab.com/products/Flag-Tag-Antibody-20543-1-AP.htm)
anti-LAMIN B : Abcam, 16048, validated in WB (0.1 μg/ml) (https://www.abcam.com/lamin-b1-antibody-nuclear-envelope-marker-ab16048.html)
anti-53BP1 : Novus Biologicals, NB100-304, validated in WB (1:5,000-1:25,000) (https://www.novusbio.com/products/53bp1-antibody_nb100-304)
anti-XRCC4 : Santa Cruz, clone C20, sc-8285, validated in WB (https://www.scbt.com/fr/p/xrcc4-antibody-c-20)

Antibodies used in Flow Cytometry studies:
anti-hCD4: Miltenyi, clone M-T466, 130-113-254, validated in Flow Cytometry (1:50) (https://www.miltenyibiotec.com/FR-en/products/cd4-antibody-anti-human-m-t466.html#gref)
anti-CD16-32: BD Biosciences, clone 2.4G2, 553142, validated in Flow Cytometry (https://www.bdbiosciences.com/en-fr/products/reagents/flow-cytometry-reagents/research-reagents/single-color-antibodies-ruo/purified-rat-anti-mouse-cd16-cd32-mouse-bd-fc-block.553142)

anti-CD19: BD Biosciences, clone 1D3, 560375, validated in Flow Cytometry (https://www.bdbiosciences.com/en-fr/products/reagents/flow-cytometry-reagents/research-reagents/single-color-antibodies-ruo/v450-rat-anti-mouse-cd19.560375)
anti-CD43: BD Biosciences, clone S7, 553271, validated in Flow Cytometry (https://www.bdbiosciences.com/en-fr/products/reagents/flow-cytometry-reagents/research-reagents/single-color-antibodies-ruo/pe-rat-anti-mouse-cd43.553271)
anti-B220: BD Biosciences, clone RA3–6B2, 557669, validated in Flow Cytometry (https://www.bdbiosciences.com/en-fr/products/reagents/flow-cytometry-reagents/research-reagents/single-color-antibodies-ruo/alexa-fluor-488-rat-anti-mouse-cd45r.557669)
anti-B220: BD Biosciences, clone RA3–6B2, 553092, validated in Flow cytometry (https://www.bdbiosciences.com/en-fr/products/reagents/flow-cytometry-reagents/research-reagents/single-color-antibodies-ruo/apc-rat-anti-mouse-cd45r-b220.553092)
anti-IgM: BD Biosciences, clone R6–60.2, 552867, validated in Flow Cytometry (https://www.bdbiosciences.com/en-fr/products/reagents/flow-cytometry-reagents/research-reagents/single-color-antibodies-ruo/pe-cy-7-rat-anti-mouse-igm.552867)
anti-CD4: BD Biosciences, clone RM4–5, 553048, validated in Flow Cytometry (https://www.bdbiosciences.com/en-fr/products/reagents/flow-cytometry-reagents/research-reagents/single-color-antibodies-ruo/pe-rat-anti-mouse-cd4.553048)
anti-CD8: BD Biosciences, clone 53-6.7, 557668, validated in Flow Cytometry (https://www.bdbiosciences.com/en-fr/products/reagents/flow-cytometry-reagents/research-reagents/single-color-antibodies-ruo/alexa-fluor-488-rat-anti-mouse-cd8a.557668)
anti-CD3e: BD Biosciences, clone 145-2C11, 553066, validated in Flow Cytometry (https://www.bdbiosciences.com/en-fr/products/reagents/flow-cytometry-reagents/research-reagents/single-color-antibodies-ruo/apc-hamster-anti-mouse-cd3e.553066)
anti-CD44: BD Biosciences, clone IM7, 560451, validated in Flow Cytometry (https://www.bdbiosciences.com/en-fr/products/reagents/flow-cytometry-reagents/research-reagents/single-color-antibodies-ruo/v450-rat-anti-mouse-cd44.560451)
anti-CD25: BD Biosciences, clone PC61, 552880, validated in Flow Cytometry (https://www.bdbiosciences.com/en-fr/products/reagents/flow-cytometry-reagents/research-reagents/single-color-antibodies-ruo/pe-cy-7-rat-anti-mouse-cd25.552880)
anti-TCRβ: eBioscience, clone H57–597, 47-5961-82, validated in Flow Cytometry (1 µg/test) (https://www.thermofisher.com/antibody/product/TCR-beta-Antibody-clone-H57-597-Monoclonal/47-5961-82)
anti-CD93 : BD Biosciences, clone AA4.1, 558039, validated in Flow Cytometry (https://www.bdbiosciences.com/en-fr/products/reagents/flow-cytometry-reagents/research-reagents/single-color-antibodies-ruo/pe-rat-anti-mouse-cd93-early-b-lineage.558039)
anti-CD23 : BD Biosciences, clone B3B4, 562929, validated in Flow Cytometry https://www.bdbiosciences.com/en-in/products/reagents/flow-cytometry-reagents/research-reagents/single-color-antibodies-ruo/bv421-rat-anti-mouse-cd23.562929)
anti-CD21 : BD Biosciences, clone 7G6, 561770, validated in Flow Cytometry (https://www.bdbiosciences.com/en-fr/products/reagents/flow-cytometry-reagents/research-reagents/single-color-antibodies-ruo/apc-rat-anti-mouse-cd21-cd35.561770)
anti-IgG1: BD Biosciences, clone X56, 550874, validated in Flow Cytometry (https://www.bdbiosciences.com/en-fr/products/reagents/flow-cytometry-reagents/research-reagents/single-color-antibodies-ruo/apc-rat-anti-mouse-igg1.550874)
anti-IgG2b: Biolegend, clone RMG2b-1, 406708, validated in Flow Cytometry (https://www.biolegend.com/fr-ch/products/pe-anti-mouse-igg2b-9652)
anti-IgG3: BD Biosciences, clone R40-82, 553403, validated in Flow Cytometry (https://www.bdbiosciences.com/content/dam/bdb/products/global/reagents/flow-cytometry-reagents/research-reagents/single-color-antibodies-ruo/553403_base/pdf/553403.pdf)
anti-Thy1.1: BD Bioscience, clone HIS51, 740044, validated in Flow Cytometry (https://www.bdbiosciences.com/en-fr/products/reagents/flow-cytometry-reagents/research-reagents/single-color-antibodies-ruo/bv421-mouse-anti-rat-cd90-mouse-cd90-1.740044)
anti-IgA: eBiosciences, clone mA-6E1, 12-4204-82, validated in Flow Cytometry (0.125 µg/test) (https://www.thermofisher.com/antibody/product/IgA-Antibody-clone-mA-6E1-Monoclonal/12-4204-82)

Eukaryotic cell lines

Policy information about cell lines

Cell line source(s)

Murine derived v-Abl/Bcl2 pro-B cell lines were generated as previously described (Lenden Hasse, H. et al. Generation and CRISPR/Cas9 editing of transformed progenitor B cells as a pseudo-physiological system to study DNA repair gene function in V(D)J recombination. J Immunol Methods 451, 71-77 (2017); Lescale, C. et al. RAG2 and XLF/Cernunnos interplay reveals a novel role for the RAG complex in DNA repair. Nat Commun 7, 10529 (2016)). Briefly, total bone marrow from 3–5-week-old mice was cultured and infected with a retrovirus encoding v-Abl kinase to generate immortalized pro-B cell lines. v-abl transformed pro-B cell lines were then transduced with pMSCV-Bcl2-puro retrovirus to protect them from v-abl kinase inhibitor-induced cell death.

CRISPR-Cas9 edited v-Abl/Bcl2 pro-B cell clones were generated as previously described (Lenden Hasse, H. et al. Generation and CRISPR/Cas9 editing of transformed progenitor B cells as a pseudo-physiological system to study DNA repair gene function in V(D)J recombination. J Immunol Methods 451, 71-77 (2017); Lescale, C. et al. Specific Roles of XRCC4 Paralogs PAXX and XLF during V(D)J Recombination. Cell Rep 16, 2967-2979 (2016)).

CRISPR-Cas9 edited CH12F3 cell clones were generated as previously described (Dev H, et al, Nature Cell Biology, 2018). The authors thank F. Alt (Harvard University, USA) for CH12F3 cells and 53bp1 knockout CH12F3 cell clones and T. Honjo (Kyoto University, Japan) for permission to use the CH12F3 cell line.

List of cell lines used in this study:

v-abl Pro-B cell lines:
12095: WT, murine-derived pro-B cells (Lescale C. et al., Nat Com. 2016)
12096: WT, murine-derived pro-B cells (Lescale C. et al., Nat Com. 2016)
GBB: WT, murine-derived pro-B cells (This study)
O38: WT, CRISPR-Cas9 knockout pro-B cell clone (This study)
9999: 53bp1-/-, murine-derived pro-B cells (Liu X. et al., PNAS. 2012)
1110: 53bp1-/-, murine-derived pro-B cells (Liu X. et al., PNAS. 2012)
BP95-2: 53bp1-/-, CRISPR-Cas9 knockout pro-B cell clone (This study)

	BP96-5: 53bp1-/-, CRISPR-Cas9 knockout pro-B cell clone (This study)<br>O32: Shld1-/-, CRISPR-Cas9 knockout pro-B cell clone (This study)<br>O37: Shld1-/-, CRISPR-Cas9 knockout pro-B cell clone (This study)<br>O44: Shld1-/-, CRISPR-Cas9 knockout pro-B cell clone (This study)<br>GBO1: Shld1-/-, murine-derived pro-B cells (This study)<br>16488: Xlf-/-, murine-derived pro-B cells (Lescale C. et al., Cell Rep. 2016)<br>X95-3: Xlf-/-, CRISPR-Cas9 knockout pro-B cell clone (Lescale C. et al., Cell Rep. 2016)<br>XO2-8: Shld1-/- Xlf-/-, CRISPR-Cas9 knockout pro-B cell clone (This study)<br>XO2-24: Shld1-/- Xlf-/-, CRISPR-Cas9 knockout pro-B cell clone (This study)<br>9x1: 53bp1-/- Xlf-/-, CRISPR-Cas9 knockout pro-B cell clone (This study)<br>9x2: 53bp1-/- Xlf-/-, CRISPR-Cas9 knockout pro-B cell clone (This study)<br><br>CH12 cell lines:<br>CH12: WT (Nakamura M. et al., International Immunology 1996)<br>O2: WT (Dev H. et al., Nat Cell Biol. 2018)<br>53bp1#1: 53bp1-/-, CRISPR-Cas9 knockout CH12 cell clone (Panchakshari R.A. et al. PNAS 2018)<br>53bp1#2: 53bp1-/-, CRISPR-Cas9 knockout CH12 cell clone (Panchakshari R.A. et al. PNAS 2018)<br>O6: Shld1-/-, CRISPR-Cas9 knockout CH12 cell clone (Dev H. et al., Nat Cell Biol. 2018)<br>O12: Shld1-/-, CRISPR-Cas9 knockout CH12 cell clone (Dev H. et al., Nat Cell Biol. 2018)<br>XR5: Xrcc4-/-, CRISPR-Cas9 knockout CH12 cell clone (This study)<br>XR1: Xrcc4-/-, CRISPR-Cas9 knockout CH12 cell clone (This study)<br>XRO6-14: Shld1-/- Xrcc4-/-, CRISPR-Cas9 knockout CH12 cell clone (This study)<br>XRO6-27: Shld1-/- Xrcc4-/-, CRISPR-Cas9 knockout CH12 cell clone (This study)<br>XR-53BP1-12: Xrcc4-/- 53bp1-/-, CRISPR-Cas9 knockout CH12 cell clone (This study)<br>XR-53BP1-22: Xrcc4-/- 53bp1-/-, CRISPR-Cas9 knockout CH12 cell clone (This study)
Authentication	All WT and knock out v-abl pro-B cells and CH12 cells were identified by PCR assays with species-specific primers, sanger sequencing and western blotting.
Mycoplasma contamination	The cell lines were not tested for mycoplasma contamination.
Commonly misidentified lines<br>(See ICLAC register)	No commonly misidentified cell lines were used in this study.

Animals and other organisms

Policy information about studies involving animals; ARRIVE guidelines recommended for reporting animal research

Laboratory animals	Mice were bred under pathogen-free (SPF) conditions and housed at ambient temperature and humidity with 12 h light/12 h dark cycles.<br><br>Shld1+/− Xlf−/− mice were bred with Shld1+/− Xlf−/− mice, and Shld1−/− Xlf+/− with Shld1−/− Xlf+/− to generate doubly deficient mice. Shld1-/- Xrcc4f/f mice were crossed to Shld1-/- CD21creTg Xrcc4f/+ to generate Shld1-/- CD21-creTg Xrcc4f/f mice.<br><br>The Xlf-/-, Atm-/-, 53bp1-/-, Xrcc4f/f and CD21-CreTg mice were used in previous studies:<br>Dong, J. et al. Orientation-specific joining of AID-initiated DNA breaks promotes antibody class switching. Nature 525, 134-139 (2015).<br>Lescale, C. et al. RAG2 and XLF/Cernunnos interplay reveals a novel role for the RAG complex in DNA repair. Nat Commun 7, 10529 (2016).<br>Sundaravinayagam D, et al. 53BP1 Supports Immunoglobulin Class Switch Recombination Independently of Its DNA Double-Strand Break End Protection Function. Cell Rep 28, 1389-1399 (2019).<br>Yan, C.T. et al. XRCC4 suppresses medulloblastomas with recurrent translocations in p53-deficient mice. Proc Natl Acad Sci U S A 103, 7378-7383 (2006).<br>Kraus, M., Alimzhanov, M.B., Rajewsky, N. & Rajewsky, K. Survival of resting mature B lymphocytes depends on BCR signaling via the Igalpha/beta heterodimer. Cell 117, 787-800 (2004).<br><br>6-17 weeks-old males and females were used.
Wild animals	The study did not involve wild animals.
Field-collected samples	The study did not involve samples collected from the field.
Ethics oversight	All experiments were performed after authorization was granted by the institutional animal care and ethical committee of Institut Pasteur/CETEA n°89 under the protocol numbers 180006/14778.

Note that full information on the approval of the study protocol must also be provided in the manuscript.

Flow Cytometry

Plots

Confirm that:

- [x] The axis labels state the marker and fluorochrome used (e.g. CD4-FITC).
- [x] The axis scales are clearly visible. Include numbers along axes only for bottom left plot of group (a 'group' is an analysis of identical markers).
- [x] All plots are contour plots with outliers or pseudocolor plots.
- [x] A numerical value for number of cells or percentage (with statistics) is provided.

Methodology

Sample preparation

For V(D)J recombination assays, purified pMX-INV v-Abl pro-B cells were treated with 3 µM of the Abl kinase inhibitor STI-571 for 72 h, washed with PBS +2% FBS and stained with hCD4 antibody at 4°C for 30 min. Cells were then washed and resuspended in PBS +2% FBS before acquisition.

For mouse analysis, single-cell suspensions of spleen, bone marrow, and thymus were washed with PBS +2% FBS and stained with cell surface markers at 4°C for 30 min. Cells were then washed and resuspended in PBS +2% FBS before acquisition.

For class-switch recombination assay with primary cells, splenic B-cells were purified from 6–8 week-old mice using magnetic CD19 beads and were stimulated with LPS (25µg/ml, Sigma Aldrich), IL-4 (10ng/ml, Miltenyi) and anti-IgD dextran (3 ng/ml, Fina Biosolutions) for IgG1, and LPS for IgG2b and IgG3 for 4 to 5 days, washed with PBS +2% FBS and stained with cell surface markers at 4°C for 30 min. Cells were then washed and resuspended in PBS +2% FBS before acquisition.

For class-switch recombination assay with CH12F3 cells, B cells were treated with anti- CD40 antibody (1 µg/ml, Miltenyi), IL-4 (20 ng/ml, Miltenyi) and TGF-β (1 ng/ml, R&D Biotech) for 3 days, washed with PBS +2% FBS and stained with cell surface markers at 4°C for 30 min. Cells were then washed and resuspended in PBS +2% FBS before acquisition.

Instrument

Samples were acquired on a BD FACS Canto II and LSR Fortessa (BD Biosciences).

Software

Samples were aquired using FACS Diva v8.0.1 and analysed using Flowjo v10.

Cell population abundance

After transfection of Cas9-GFP and guide RNA plasmids, GFP positive cells were sorted into single cell for the selection of knock out clones.

Gating strategy

For V(D)J recombination assays, live cells were gated using FSC-A/SSC-A, then single cells were gated using FSC-H/FSC-W. V(D)J recombination levels were scored as the percentage of GFP positive cells among total hCD4 positive cells.

For mouse analysis, live lymphocytes cells were gated using FSC-A/SSC-A, then single cells were gated using FSC-H/FSC-W. Bone marrow B lineage cell populations were identified based on the expression of the following markers: pro-B (B220lo CD43+ CD19+ IgM−), pre-B (B220lo CD43− CD19+ IgM−), immature B cells (B220lo IgM+), recirculating B cells (B220hi IgM+). T lineage cell populations from the thymus were identified based on the expression of the following markers: double-negative (DN) cells (CD4−CD8−), DN1 (CD4−CD8−CD44+CD25−), DN2 (CD4−CD8−CD44+CD25+), DN3 (CD4−CD8−CD44−CD25+), DN4 (CD4−CD8−CD44−CD25−), double-positive (DP) cells (CD4+CD8+) and single-positive (SP) cells (CD4+CD8− and CD4−CD8+). Lymphocytes from the spleen were identified based on the expression of the following markers: total B cells (CD19+IgM+), Marginal Zone B cells (B220+CD93-CD23-CD21high), Follicular B cells (B220+CD93-CD23+CD21+) and T cells (CD3+TCRβ+).

For class-switch recombination assays, live cells were gated using FSC-A/SSC-A, then single cells were gated using FSC-H/FSC-W. Class-switch recombination levels were scored as the percentage of either IgG1, IgG2b, or IgG3 positive cells among total CD19 positive cells; or IgA positive cells among total single cells.

- [x] Tick this box to confirm that a figure exemplifying the gating strategy is provided in the Supplementary Information.
